# Therapeutic advances of targeting receptor tyrosine kinases in cancer

**DOI:** 10.1038/s41392-024-01899-w

**Published:** 2024-08-14

**Authors:** Ciprian Tomuleasa, Adrian-Bogdan Tigu, Raluca Munteanu, Cristian-Silviu Moldovan, David Kegyes, Anca Onaciu, Diana Gulei, Gabriel Ghiaur, Hermann Einsele, Carlo M. Croce

**Affiliations:** 1 Medfuture Research Center for Advanced Medicine, Iuliu Hatieganu University of Medicine and Pharmacy, Cluj-Napoca, Romania; 2 Department of Hematology, Iuliu Hatieganu University of Medicine and Pharmacy, Cluj Napoca, Romania; 3 Department of Hematology, Ion Chiricuta Clinical Cancer Center, Cluj Napoca, Romania; 4 Academy of Romanian Scientists, Ilfov 3, 050044 Bucharest, Romania; 5 Department of Leukemia, Sidney Kimmel Cancer Center at Johns Hopkins, Johns Hopkins University School of Medicine, Baltimore, MD USA; 6 Universitätsklinikum Würzburg, Medizinische Klinik II, Würzburg, Germany; 7 Department of Cancer Biology and Genetics and Comprehensive Cancer Center, The Ohio State University, Columbus, OH USA

**Keywords:** Structural biology, Cancer genetics

## Abstract

Receptor tyrosine kinases (RTKs), a category of transmembrane receptors, have gained significant clinical attention in oncology due to their central role in cancer pathogenesis. Genetic alterations, including mutations, amplifications, and overexpression of certain RTKs, are critical in creating environments conducive to tumor development. Following their discovery, extensive research has revealed how RTK dysregulation contributes to oncogenesis, with many cancer subtypes showing dependency on aberrant RTK signaling for their proliferation, survival and progression. These findings paved the way for targeted therapies that aim to inhibit crucial biological pathways in cancer. As a result, RTKs have emerged as primary targets in anticancer therapeutic development. Over the past two decades, this has led to the synthesis and clinical validation of numerous small molecule tyrosine kinase inhibitors (TKIs), now effectively utilized in treating various cancer types. In this manuscript we aim to provide a comprehensive understanding of the RTKs in the context of cancer. We explored the various alterations and overexpression of specific receptors across different malignancies, with special attention dedicated to the examination of current RTK inhibitors, highlighting their role as potential targeted therapies. By integrating the latest research findings and clinical evidence, we seek to elucidate the pivotal role of RTKs in cancer biology and the therapeutic efficacy of RTK inhibition with promising treatment outcomes.

## Introduction

Beginning in the early 1950s, notable progress was achieved in the field of cellular biology through the discovery of receptor tyrosine kinases (RTKs). Although identified as the receptors for insulin and epidermal growth factor (EGF), RTKs subsequently became the primary focus for understanding cellular signaling systems.^1,2^ During this time, nerve growth factor and EGF were discovered and found to have significant impacts on the development of neurons and the proliferation of cells, both in living organisms and in laboratory settings.

By the 1960s, extensive research on insulin had deepened understanding of the interactions of its receptor. Scientists performed thorough examinations of insulin’s interaction with its receptor on cells or solubilized receptor preparations utilizing radiolabeled insulin. These findings confirmed the ligand-binding properties and introduced the notion of negative interaction in insulin binding. The understanding of this concept was further intensified during the 1970s. The researchers mapped the precise locations on the surfaces of cells where EGF binds and made a connection between the phosphorylation of proteins on tyrosine residues and the signaling within cells, as well as the potential processes that may lead to the development of cancer.^3^ During this decade, key features of receptors were identified, such as ligand-dependent down-regulation and desensitization via internalization and degradation, observed in both the insulin receptor and EGFR.^4^ By the early 1980s, it was well-recognized that certain receptors function as ligand-activated protein tyrosine kinases. These discoveries highlighted the role of RTKs in regulating cellular development, vital physiological functions, and cancer development, significantly enhancing our understanding of cellular mechanisms.^5,6^

The RTK family encompasses a diverse array of cell surface receptors that respond to growth factors, hormones, and cytokines, mediating a wide range of fundamental cellular and metabolic signaling pathways.^7^ The common denominators of this receptor family consist of the conserved structural domains, namely, the extracellular ligand-binding domain, the transmembrane helix, and the intracellular tyrosine kinase domain. The extracellular domain of RTKs is a dynamic region that governs ligand binding, receptor activation, and subsequent signaling cascades, making it a key determinant of RTK function and cellular responses.^8^ Ligand specificity and binding affinity are crucial properties in influencing downstream signaling events.^9^ Specifically, it consists of distinct structural elements, such as immunoglobulin-like domains, fibronectin type III-like repeats, EGF-like domains, and cysteine-rich regions, which contribute to the classification of RTKs into different families based on their structural extracellular characteristics.^10^ As such, the number, combination, and arrangement of these domains vary significantly among different RTK families, conferring unique ligand-binding capabilities and regulatory properties to each receptor.^11^ First, the immunoglobulin-like domains (Ig-like) typically exhibit a sandwich-like structure composed of two β-sheets stabilized by a disulfide bond.^12^ Named based on their structural similarity to immunoglobulin molecules, they play a crucial role in ligand biding and dimerization. Next, the cysteine-rich domains specific to some classes of RTKs, define loop-rich compact structures that improve the conformational stability of the domain, at the same time influencing the ligand specificity and binding affinity. The fibronectin type III (FN3) repeat is typically comprised of about 90 amino acids and adopts a compact domain structure known for its β-sandwich configuration, also influencing the specific interaction capabilities of FN3-containing RTKs. Lastly, EGF-like repeats are another significant structural motif found in a variety RTKs.^13^ Named after their identification in the EGF, these repeats play an important role in ligand binding and receptor activation, influencing the signaling pathways in the context of RTKs. The intracellular helix within the kinase domain of receptor tyrosine kinases is a notable structural element. It forms an α-helical structure from a sequence of amino acids and contributes to the overall function and regulation of the kinase.^14^ Positioned within the kinase domain, this helix aids in maintaining the enzyme’s conformation and is involved in adenosine triphosphate (ATP) binding. Its interactions with other parts of the kinase domain, such as the activation loop, are part of the mechanism controlling the kinase’s activity.^15^ When the kinases are activated, the intracellular helix often undergoes a shift in position, aligning the required residues allowing catalytic activity. This helix also influences substrate access to the active site and might have a role in interactions with regulatory molecules. The intracellular domain of RTKs is the cornerstone in cellular signal transduction. At the heart of this domain lies the tyrosine kinase domain, an enzymatic center that catalyzes the phosphorylation of specific tyrosine residues on target proteins via ATP.^10^ The intracellular domain also encompasses regulatory regions, such as the juxta-membrane domains, which can inhibit kinase activity in the absence of a ligand, and C-terminal tails that often contain multiple tyrosine residues.^16^ These residues, upon phosphorylation, serve as docking sites for adaptor and effector proteins, crucial for signal propagation. The process begins with ligand binding to the RTK’s extracellular domain, triggering receptor dimerization and subsequent autophosphorylation. This autophosphorylation of specific tyrosine residues within the intracellular domain creates binding sites for proteins with Src homology 2 (SH2) or phosphotyrosine binding (PTB) domains.^17^

**Fig. 1:**
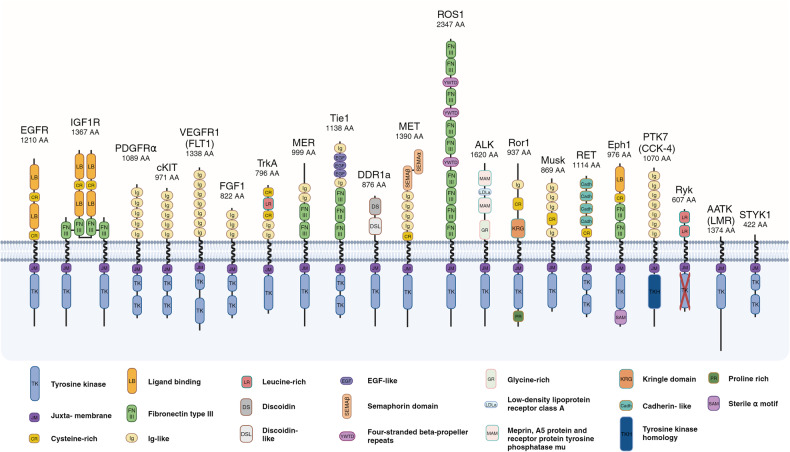
Structure of the 20 Receptor Tyrosine Kinase Classes. The RTKs structure differs from one receptor to another, with several similarities and differences mostly at the extracellular and intracytoplasmic domains as depicted from left to right in all 20 RTKs classes. Images created with BioRender.com

RTKs are grouped into 20 families, based on their amino acid sequence similarities and structural characteristics in their extracellular domains, leading to members within a family binding to similar or same ligands.^18^ Fig. 1 provides a visual representation of the different domains found in RTKs, highlighting the structural variations that contribute to their diverse roles in cellular signaling.

**Fig. 2:**
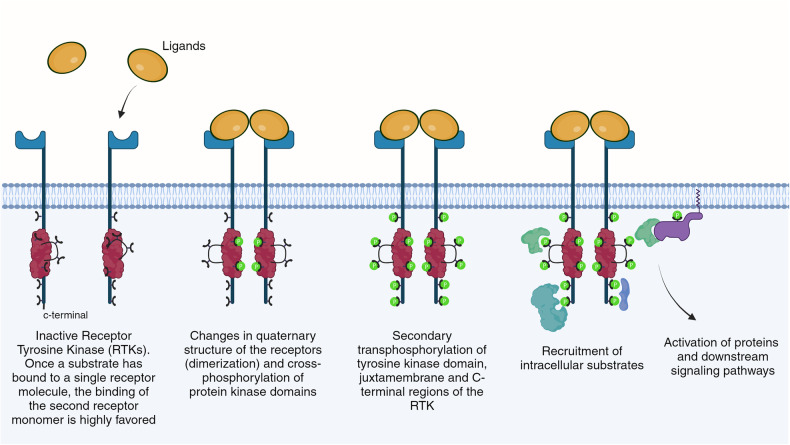
Activation and Intracellular Signaling Mechanisms of Receptor Tyrosine Kinase. Depicted from left to right, the Ligands are binding to the monomer RTKs and trigger the dimerization with a cross-phosphorylation of the protein kinase domains. Further secondary transphosphorylation of the tyrosine kinase domains, juxtamembrane and c-terminal regions occur in the RTKs, which will further create the proper conditions for the recruitment of the intracellular substrates that will further lead to the activation of the key proteins in the downstream signaling pathways. Images created with BioRender.com

The activation of RTKs is a multifaceted process, influenced by a delicate balance between external ligand availability and intrinsic receptor conformational dynamics. At the molecular level, RTK activation is not a uniform event but rather a confluence of diverse regulatory mechanisms that reflect the complex biological systems they modulate. The process initiates with the extracellular domain of RTKs, which, upon binding to specific ligands such as growth factors, undergoes structural alterations, a prerequisite for the trans-autophosphorylation of tyrosine residues within the intracellular kinase domains (Fig. 2).

As previously described, the specificity of ligand binding is crucially determined by the cumulative properties of the extracellular domain, ensuring that the signal is initiated only in response to the appropriate extracellular cues (i.e., ligand).^19^ Next, receptor dimerization consists of the pairing of RTK molecules because of ligand binding, facilitating the cross-phosphorylation of tyrosine residues in their intracellular domains.^20^ Such phosphorylation is essential for activating the kinase function of the receptors, not only by activating the RTKs per se but also by generating binding sites for various intracellular signaling proteins.^21^ Trans-autophosphorylation, where the kinase domains of the dimerized receptors become strategically aligned, facilitates each kinase domain to phosphorylate specific tyrosine residues on its partner in the dimer.^22^ This trans-autophosphorylation is a key event, as it activates the kinase domains, marking a transition from a dormant state to an active one. Subsequently, the phosphorylated tyrosines on the RTKs transform into critical docking sites for various intracellular signaling proteins. These proteins, often equipped with SH2 or PTB domains, have a high affinity for the phosphorylated tyrosines.^23,24^ Their binding to these activated sites on the RTKs is not just a mechanical linkage; it’s the initiation of a complex network of downstream signaling pathways.

## Classification of RTK protein families

RTK subfamilies and their members, along with the corresponding discovered ligand, emphasizing on the specific functions and roles of the receptors in cell development, growth and proliferation, metabolism modulation, cell cycle, epithelial to mesenchymal transition and many other physiological and physiopathological involvement are highlighted in Table 1.

**Table 1:** Classification of RTK subfamilies based on kinase domain sequence

RTK subfamilies	Members	Ligands	Function	Ref
EGFR/ErbB	EGFR, ErbB2, ErbB3, ErbB4	EGFR, ErbB2, B3,B4	Cell growth, differentiation	^61,62^
FGFR	FGFR1, FGFR2, FGFR3, FGFR4	FGF-1-10, FGF-16-23	Stimulating cell growth, differentiation, migration, angiogenesis and cell survival	^822^
IR	INSR, IGF1R	Insulin, IGF-I, IGFII	Facilitating the transport of glucose intro cells, enhancing glycogen synthase, controlling metabolic processes like protein and lipid production; cell growth	^823^
PDGFR	PDGFRα, PDGFRβ, CSF1R/Fms, Kit/SCFR, Flt3/Flk2	PGDF-A, PGDF-B, PGDF-C, PGDF-D, IL-34, CSF-1, SCF and FLT3LG	Regulates cell growth, proliferation and differentiation	^134^
VEGFR	VEGFR1, VEGFR2, VEGFR3	VEGF-A, VEGF-B, VEGF-C, VEGF-D, PIGF	Regulating angiogenesis development	^824^
HGFR/Met	Met, MST1 R, RON	HGF, HGF-like/macrophage stimulating protein (MSP)	MET signaling mediates cellular motility, invasion, angiogenesis and cellular growth	^825^
MuSK	MUSK	LRP4 Agrin	Has a central role in neuromuscular junction formation.	^826^
LTK/ALK	LTK, ALK	ALKAL1,2 Pleiotrophin, midkine	Regulate pathways involved in cell growth and differentiation	^827,828^
ROS1	ROS	Unknown yet	Abnormal expression and mutated versions of ROS kinase are detected in several malignancies	^134^
RET	RET9,43,51	GFLs, Neurturin, persephin, artemin	Cell proliferation, differentiation and survival	^270^
ROR	ROR1,2	Wnt-1, -3, -5a	Regulate cell division, proliferation and differentiation, during embryogenesis, while in adult tissue is less expressed, except tumor cells	^829,830^
EPHR	EPHA 1,2,3,4,5,6,7,8, 10, EPHB1,B2,B3,B4,B6	Ephrin A1,A2,A3,A4,A5,B1,B2,B3,B4,B5	Activation, migration, adhesion and proliferation of immune cells	^831^
RYK	Ryk	Wnt-1, -3a, -5a	Modulates EMT process, migration and proliferation	^830,832^
CCK4-PTK7	CCK4-PTK7	Wnt ligands	Neural tube formation	^134^
NGFR/Trk	TRKA, TRKB, TRKC	Nerve growth factor, neutrophin NT-3, brain-derived neurotrophic factor (BDNF), NT-4, NT5	Involved in neuronal survival and differentiation.	^833^
AXL/TAM	AXL, MER, TYRO3	Gas6, PROS1, tubby-elated protein (Tulp)-1	Implicated in the phagocytic process of removing apoptotic cells in adult organisms	^834,835^
TIE	Tie1,2	Angpt-1, -2, -4	Regulating embryonic development	^836^
DDR family	DDR1,2	Collagen type I,II,III,IV and type X	DDR1 development of the mammary gland DD2 elongation and growth of long bones	^837,838^
LMR	LMR 1 (AATK), LMR 2 (AATYK2), LMR 3 (AATYK3)	unknown	Regulate protein transport, endosome sorting, vesicle secretion and protein localization; Involved in cell proliferation	^58,409^
STYK1	STYK1	unknown	Epithelial to mesenchymal transition, autophagy; cell development processes	^58^

## Physiological roles of RTKs

### Key signaling mechanisms of RTKs

Following the recruitment of signaling proteins to the phosphorylated and activated RTKs, a series of intricate signal transduction cascades is initiated, each targeting specific cellular functions. Prominently, the Mitogen-Activated Protein Kinase (MAPK) pathway is activated, playing a central role in regulating gene expression and orchestrating cellular processes like proliferation and differentiation. Concurrently, the Phosphoinositide 3-Kinase/Protein Kinase B (PI3K/Akt) pathway is mobilized, which is crucial for controlling various aspects of cell survival, growth, and metabolism.^25^ Another key pathway activated is the Phospholipase C gamma (PLCγ) pathway, influential in modulating calcium signaling and cytoskeletal reorganizations.^26^

### Termination of RTK signaling

**Fig. 3:**
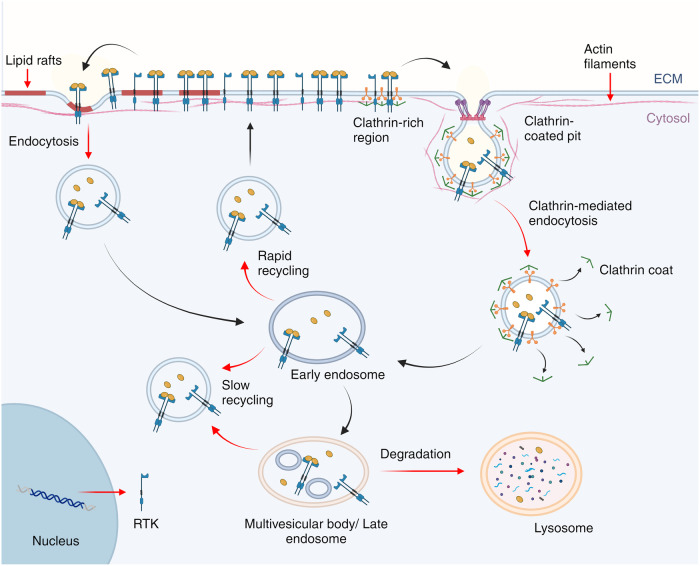
Termination of RTKs signaling—endocytosis of signaling and Endocytic Trafficking. The red arrows in the figure illustrate variations in the endocytic rate and pathway selection, which are determinant factors for the modulation of RTK surface expression and downstream signaling, potentially driving oncogenic processes. Images created with BioRender.com

To maintain cellular homeostasis and prevent overactivation of these pathways, a set of regulatory mechanisms came into play. These include dephosphorylation of the RTKs by phosphatases, internalization and degradation of the receptors, and feedback inhibition from downstream signaling components.^27,28^ This regulatory phase is essential for ensuring that the signaling is transient and contextually appropriate, providing a fail-safe against uncontrolled or prolonged activation that could lead to pathological conditions. However, in the context of cancer, dysregulation of these regulatory pathways is common. Altered plasma membrane domains and endocytic trafficking in tumor cells lead to aberrant RTK clustering and signaling^29^ (Fig. 3).

### Role in cellular growth and proliferation

RTKs are instrumental in regulating cellular growth and proliferation. Upon activation by ligand binding, they initiate a cascade of intracellular signaling, predominantly through the Ras/MAPK pathway, leading to the transcription of genes that drive cell cycle progression. This signaling mechanism is crucial for the controlled growth of cells, ensuring that proliferation occurs in response to appropriate external stimuli. Dysregulation of this process, often seen in the overactivation of RTKs, is a hallmark of various cancers, underlining the critical role of RTK signaling in maintaining normal cell growth and division.^30–35^

### RTKs in cellular differentiation and development

RTKs are vital in guiding cellular differentiation and development. They are key players in embryonic development, influencing cell fate decisions and tissue formation. For instance, Fibroblast Growth Factor Receptors (FGFRs) have a well-established role in limb development, while Epidermal Growth Factor Receptors (EGFRs) are crucial in neural development. Through binding with specific ligands, RTKs activate signaling pathways that lead to the differentiation of cells into specialized types, crucial for the proper formation and function of diverse tissues and organs.^36^

### RTKs in metabolism regulation

The metabolic profiles of tumor cells are different from normal cells; thus, tumor cells tend to rewire the metabolism to support tumor progression due to the high metabolic demands.^37^ Resistance to therapy can occur also due to the metabolic adaptations suggesting that the cellular metabolism can be crucial in tumorigenesis.^38^ RTKs activation can modulate different metabolic pathways and RTKs driven metabolic reprogramming could lead to different metabolic vulnerabilities that could be targeted, for example, the lactate production can fuel the TCA cycle generating energy in FGFR aberrant cancer cells, serine synthesis can be used for nucleotide biosynthesis and redox homeostasis of EGFR constitutively activated tumors. Jin et al., showed that the RTK involvement in metabolism can induce metabolic reprogramming and provide distinct metabolic vulnerabilities that can be exploited.^39^ The interplay between RTKs and other metabolic pathways underscores their importance in maintaining metabolic balance within the body.

### RTKs in cell survival and apoptosis

The balance between cell survival and programmed cell death (apoptosis) is tightly regulated by RTK signaling. By activating pathways like PI3K/Akt, RTKs promote cell survival and inhibit apoptotic pathways. This protective role is essential for normal cellular function and response to stress. However, aberrant activation of these pathways can lead to uncontrolled cell survival, contributing to the development of cancer. RTKs, therefore, play a dual role in maintaining cellular health, promoting survival under normal conditions, and facilitating apoptosis when cells are damaged or no longer needed. The pathways and kinetics of RTK endocytic trafficking, molecular mechanisms underlying sorting processes, and examples of deviations from the standard trafficking itinerary in the RTK family are discussed in the literature.^40^ Additionally, overexpression of RTK proteins or functional alterations caused by mutations in the corresponding genes or abnormal stimulation by autocrine growth factor loops contribute to constitutive RTK signaling, resulting in alterations in the physiological activities of cells.^41^

The cystine-rich domains of variable length are commonly found in RTKs, and multiple RTKs contain Ig domains, with the ectodomain of certain families consisting solely of Ig domains. The role of alternative splicing of RTKs in tumor progression and response to therapies, with a special focus on major RTKs that control proliferation, survival, and angiogenesis, has been discussed in the literature.

## RTK dysregulation and cancer connections

The regulation of protein tyrosine phosphatase (PTP) activity plays a significant role in modulating RTK signaling, by acting concurrently.^27^ The ligand-induced inhibition of PTPs, which conventionally serve to dephosphorylate and deactivate RTKs, results in the prolonged activation of the receptor, thereby amplifying downstream signaling pathways. Moreover, certain RTKs exhibit ligand-independent activation, primarily driven by genetic mutations or overexpression.^19^ This constitutive activation can lead to aberrant signaling pathways, often implicated in pathological conditions such as oncogenesis.^42^

**Fig. 4:**
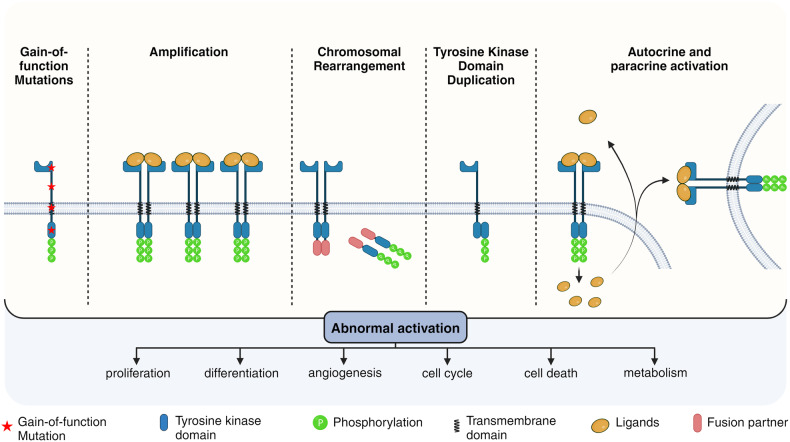
RTKs dysregulation mechanisms. The dysregulation of RTKs may occur by a gain-of -function mutation, an amplification, chromosomal rearrangements, TK domain duplication or by an autocrine or paracrine activation (left to right). The dysregulations are generating abnormal activation of the RTKs which will be translated into enhanced proliferation, differentiation or angiogenesis, same as into a dysregulated cell cycle and metabolism. Images created with BioRender.com

Cell communication with the microenvironment involves different paths and membrane receptors that are triggered by different ligands and modulate important pathways. Key biological processes are regulated by ligand-receptor binding, such as cell proliferation, differentiation, migration, cell death mechanisms and others. Tumor cells grow faster than normal cells and some stimuli can be the excess of growth factors in the microenvironment, increased number of receptors for the ligands, or there may be mutations and rearrangements in the chromosomes resulting into different protein structure.^43,44^ All RTKs consist of an extracellular region with the ligand-binding domain that is linked to the intracellular protein kinase through a transmembrane domain.^45,46^ RTKs are involved in multiple biological pathways such as differentiation, migration, survival, or apoptosis, thus any abnormality in the RTKs may induce downstream changes and dysregulate biological processes. RTKs can be dysregulated via five main mechanisms: overexpression, TK (tyrosine kinase) domain duplication, autocrine and paracrine activation, genomic rearrangements, and gain/loss of function mutations ^17^ (Fig. 4).

Due to their importance in cell growth signaling, RTKs play a crucial role in the development and progression of various malignancies. Their significance stems from their ability to trigger the intracellular signaling cascades and influence the key cellular processes such as migration, differentiation, proliferation, and survival. In cancer, many tumor cells exhibit oncogenic addiction to RTKs, and their survival is supported by the RTKs activation.^47–49^ The addiction to oncogenes has been described in 2002, highlighting the fact that tumor cells tend to sustain their survival depending on specific oncoproteins, overcoming the genetic lesions that occur in tumor cells due to their highly proliferative state.^50^ Some mutations in the RTKs are considered “*Driver mutations*” and promote a fast cell growth and sustain survival, together with the gene amplification or chromosomal rearrangements.^51,52^ The RTK activity is well controlled in normal cells, however, the receptors undergo structural changes leading to their overactivation due to a series of factors such as mutations, overexpression, or autocrine/paracrine stimulation. The structural changes or their increased density om the cell membrane increases their affinity to the ligands and overstimulates the downstream signaling.^53–55^

Pathological signaling outcomes arise when RTKs undergo abnormal activation. The oncogenic activation through different mechanisms generates an abnormal and overstimulated signaling via the receptor, increasing the proliferation and survival of tumor cells. RTKs abnormal activation is one of the cancer characteristics which makes RTKs potent targets for therapeutic intervention with specific inhibitors.^54^ RTK inhibitors can modulate different immunosuppressive cell such as tumor-associated macrophages, regulatory T cells and myeloid-derived suppressor cells that are localized in the tumor microenvironment, thus the immunosuppressive cells can become useful in the combat against tumor cells.^56,57^

EGFRs, VEGFRs, PDGFRs, FGFRs, ROR1, ROR2, and other RTKs accumulate a series of modifications which trigger their activation and lead to a metabolic reorganization in tumor cells thus increasing their tumorigenicity.^58,59^ RTKs overexpression was spotted in solid and hematological malignancies, contributing to the enhanced cell proliferation, differentiation, migration, and cell death regulation.^60^

Further discussions will point out the involvement of RTK subclasses in biological processes and cancer development, summarizing their role in different malignancies, the mechanism of action and the mutations that may occur in the genes encoding RTK proteins and highlighting their oncologic roles.

### Deregulation of EGFR in cancer

EGFRs, noted for their high affinity for epidermal growth factors, play a crucial role in cell proliferation and survival by activating Ras/MAPK, PI3K/Akt, and JAK/STAT pathways. Their modulation of critical biological processes makes them potential targets for cancer therapy.^61,62^

Discovered in 1960s, EGF was described as pro differentiation and growth stimulatory protein when binding to its receptor.^63^ The purified EGFR had 170 kDa and when binding to the ligand induces receptor clustering. Molecular cloning of the EGFR revealed the similarity with v-erbB oncogene, furthermore, three related members of the receptor family were discovered ErbB2, ErbB3, and Erb4.^64–66^

EGFR binds to EGF and Tumor Necrosis Factor-alpha ligands and control cell growth, differentiation and proliferation. The EGFR family consists of four members: ErbB-1–HER1/epidermal growth factor receptor; ErbB-2–HER2; ErbB-3–HER3, and ErbB-4–HER4.^67,68^ EGFR mutations and upregulation drive cancer progression, highlighting EGFR as a promising therapeutic target.^69^

The ErbB family (EGFR, ErbB-3, and HER2) drives cancer proliferation and survival by activating Ras/MAPK, PI3K/Akt, and JAK/STAT pathways. Notably, HER2, prominent in breast cancer (BC), represents an important target for targeted therapies. Unlike other family members, HER2 lacks a known ligand, but is known that its dimerization activates the receptor and triggers the downstream pathways. HER2 overexpression in BC makes it a good target for therapies with Herceptin and other molecules.^70^

ErbB3, or HER3, is another member of the ErbB receptor family. While it has a lesser kinase activity, it forms heterodimers with other ErbB members, particularly HER2, to activate signaling pathways. ErbB3 is involved in activating the PI3K/Akt pathway, playing a significant role in cancer development and progression. Its role in drug resistance and cancer progression has made it a target of interest in oncological research.

ErbB4, or HER4, is involved in various developmental and physiological processes. Like other ErbB receptors, it activates the Ras/MAPK, PI3K/Akt, and JAK/STAT pathways. ErbB4’s role extends beyond oncology into neurological development, making it a subject of interest in both cancer therapy and neurobiology.

The EGFR family of receptor tyrosine kinases, consisting of four members (ErbB-1/HER1, ErbB-2/HER2, ErbB-3/HER3, and ErbB-4/HER4), plays crucial roles in cell growth, differentiation, and tumor migration regulation.^71^ The first discovered ErbB receptor is EGFR for which was first described the relationship between overexpression and cancer development.^72^ Alterations in ErbB family members were found to be correlated with the progression of numerous cancers such as ovarian, esophageal, laryngeal, breast, lung, prostate cancer, and melanoma.^73–79^

*EGFR*, an early oncogene, is a key target in clinical oncology, frequently activated by mutations or overexpression across human cancers, notably in pancreatic adenocarcinoma with poor prognosis, and lung and colon cancers with detected mutations.^80–85^ Table 2 summarizes the information regarding mutations and their role in different diseases.

**Table 2:** Overview of EGFR mutations and their role in cancer progression

Receptor	Gene family/symbol	Disease	Mechanism	Mutation	Oncogenic/role	Reference
EGFR	*EGFR*	NSCLC	Affect ligand‐receptor binding	L858R	Likely oncogenic	^90,839^
EGFR	*EGFR*	NSCLC, anaplastic astrocytoma, colorectal cancer (CRC), renal cell carcinoma (RCC)	Activating mutation	L861Q	Resistance to first generation of EGFR inhibitors	^90,839–841^
EGFR	*EGFR*	NSCLC	Activating mutation	G719A	Likely oncogenic	^90,841^
EGFR	*EGFR*	NSCLC	Activating mutation	S768I	Likely oncogenic	^90,839,841^
EGFR	*EGFR*	NSCLC	Activating mutation	L833F	Likely oncogenic	^90,841^
EGFR	*EGFR*	NSCLC	Activating mutation	E796_A750del	Likely oncogenic	^90,841^
EGFR	*EGFR*	NSCLC	Unknown	L747_E749del	Likely oncogenic	^90^
EGFR	*EGFR*	NSCLC	Unknown	E709_T710delinsD	Likely oncogenic	^90^
EGFR	*EGFR*	NSCLC	Unknown	T751_E758del	Likely oncogenic	^90^
EGFR	*EGFR*	Glioblastoma multiform (GBM)	Affect ligand‐receptor binding	A289V	Oncogenic	^90^
EGFR	*EGFR*	GBM	Affect ligand‐receptor binding	A289D	Likely oncogenic	^90^
EGFR	*EGFR*	GBM	Affect ligand‐receptor binding	A289N	Likely oncogenic	^90^
		GBM	Affect ligand‐receptor binding	A289I	Likely oncogenic	^90^
EGFR	*EGFR*	GBM	Affect ligand‐receptor binding	A289T	Likely oncogenic	^90^
EGFR	*EGFR*	NSCLC	De novo or germline/Increases affinity to ATP	T790M	Oncogenic	^87,839,842^
EGFR	*EGFR*	NSCLC	De novo mutation/Activate EGFR signaling and cross-connected pathways	C797S	Likely oncogenic	^87^
EGFR	*EGFR*	NSCLC	De novo mutation/Activate EGFR signaling and cross-connected pathways	G796D	Likely oncogenic	^88^
EGFR	*EGFR*	NSCLC	Affect ligand‐receptor binding	L718Q	Likely oncogenic	^88^
EGFR	*EGFR*	NSCLC, acute myeloid leukemia (AML), pancreatic adenocarcinoma	unknown	R831H	Unknown	^839,841^

In non-small cell lung cancer (NSCLC), T790M is a very common point mutation was detected in 60% of patients with EGFR TKI resistance.^86,87^ C797S mutations were described as responsible for acquired resistance to third generation EGFR-TKI and was found in 40% of patients with mutant NSCLC with T790M mutation. Several activation mutations in *EGFR* gene are well known at the diagnostic (Exon 19 deletion, L858R, L861Q, S781I, G719A, G719C, G796D, L718Q, L844V, and T790M).^87,88^

*EGFR* mutations are frequently discovered in NSCLC, according to Fu et al. the four generations of EGFR-TKIs are efficient if different mutation status of NSCLC cells.^89^ Tumors with single mutation (Ex19del/L858R; T790M and C797S) can be targeted by all four generations of EGFR-TKIs; Double mutant cells are sensitive to all EGFR-TKIs except 2nd generation while cell with triple mutant status are sensitive only to the 4th generation of EGFR-TKIs.

According to Liu et al., *EGFR* gene mutations frequency was 2.8% for all tumor samples and 2.4% for all samples collected from patients including 32 types of tumors.^90^ The most common tumors that had *EGFR* mutations were glioblastoma multiforme (GBM) with 26.8%, lung adenocarcinoma (LUAD) with 14.4%, diffuse large B-cell lymphoma with 8.3% and skin cutaneous melanoma (SKCM) with 6.5%; on the other hand, patients with uveal melanoma, thyroid carcinoma, kidney chromophobe cell carcinoma or thymoma showed almost undetectable *EGFR* mutations.^90^ The 289aa in the Furin-like domain of EGFR was the most frequently mutated position, detected in 27 samples—A289D, A289N, A289I, A289T, A289V, and A289Rfs*9. These mutations were almost exclusively present in GBM samples, and none of these mutations are yet known to be potential targets.^90^ Mutations in the GF_recep-IV domain were detected in GMB and esophageal squamous cell carcinoma (ESCC) (G598V and G598E), both mutations being related to ligand-receptor binding disfunctions and are treated as oncogenic mutations. In LUAD most mutations were detected in the Pkinase_Tyr domain, in positions 858aa (L858R) and 747-750aa (E746_A750del, L747_E749del and L747_T751del).^90^

*EGFR* mutations are divided into seven levels, depending on the clinical targeted therapy implication, with mutations included in level 1 and R1 indicated as targetable, according to Food and Drug Administration.^91,92^ All level 1 mutations are detected in NSCLC – 28 mutations in LUAD and 2 in lung squamous cell carcinoma and concentrated in exons 19-21 - L858R, L861Q, G719A, S768I, L833F, E796_A750del, L747_E749del, E709_T710delinsD, L747_T751del, and T751_E758del.^90^

Lung cancer, head and neck cancer and esophagus carcinoma have several commonalities in terms of EGFR with increased expression of *EGFR*, high frequency of *EGFR* amplification and low indel mutations. Also, targeted therapy in the case of these three cancers shows promising efficacy. The correlation between *EGFR* abnormalities and treatment benefits underlines the importance of molecular profiling and the detection of biomarkers for a better selection of treatment.^90^

Details regarding the EGFR receptors, the genes that encode their proteins and the disease in which the EGFR receptors are involved, with the mutations that are likely oncogenic or not are presented in Table 2.

### Deregulation of FGFRs in cancer

Fibroblast growth factor receptors (FGFRs) are integral to developmental processes, with a specific binding affinity to fibroblast growth factors. They activate the Ras/MAPK, PI3K/Akt, and PLCγ pathways, affecting a range of cellular activities including cell division, growth, migration, and angiogenesis. Mutations or dysregulations in FGFRs are associated with various developmental disorders and cancers.^43,93^

The activation of the signaling pathway of FGFRs is mainly triggered by the binging of fibroblast growth factors (FGFs) and the subsequent dimerization of the receptors, which leads to intracellular kinase trans autophosphorylation.^94^ Moreover, FGFRs can be triggered in a manner that does not require a specific ligand, such as when the FGFRs gene fuses with other genes that are constantly expressed due to chromosome translocation.^95^ In a comprehensive analysis using NGS across a diverse range of tumor samples, FGFR mutations were found to be a common occurrence in cancers characterized by FGFR gene abnormalities. For instance, FGFR1 amplifications were notably prevalent in breast and lung cancer, while FGFR2 mutations were frequently in endometrial and gastric cancers (GCs). Also, FGFR mutations, including S249C hotspot mutations, were common in bladder cancer samples.^96^

FGFR comprise four genes and include seven different receptors that are differentially activated by one of the fibroblast growth factor ligands. Four transmembrane receptors are identified (FGFR1-4) and when binding their ligands, the receptors dimerize and activate downstream pathways that regulate proliferation, survival, angiogenesis and differentiation.^96–98^

Aberrations in *FGFR1-4* genes include single-nucleotide variants (SNVs), gene fusions and rearrangements, or copy number amplifications. Due to the increased frequency of alterations in *FGFR* genes, in solid and hematological malignancies, a molecular diagnostic for accurate detection of these aberrations may indicate which therapy is better.^98–101^

SNVs can induce constitutive activation of the FGFRs increasing their affinity to ligands or over activate it. *FGFR1* SNVs are rare, with N546K and K656E as the most common mutations identified, and with an unclear consequence, S125L mutation was identified in gallbladder and BC.^102,103^ The majority of SNVs were identified in *FGFR2*, which are related to NSCLC, GC and endometrial cancer. The transmembrane mutations Y375C and C382Y, plus the extracellular domain mutations S252W, W290C and P253R are more frequent than the kinase domain mutations N549H/K and K659E, according to Helsten et al.^96^ The most frequent SNVs in *FGFR3* are R248C and S249C in the extracellular domain and G370C and Y373C in the transmembrane domain, with reports in urothelial carcinomas.^104,105^ Last, but not least, SNVs in *FGDR4* are notable in rhabdomyosarcoma, with V550E and N535K contributing to autophosphorylation of the receptor, while Y367C was identified in MDA-MB453 BC cell line.^106,107^

In FGFR3, K650 and G697 were identified as hotspots for mutations in cancers. The most frequent amino acid changes at K650 were E and M, and N, Q, and T were the least frequent observed. Also, amino acid replacement in N540 position was detected for K, S, D, and H. One frequent mutation specific for FGFR3 was G697C replacement.^108^

*FGFR* gene fusions can appear due chromosomal rearrangements or translocations, increasing the receptor dimerization or dysregulating the expression of *FGFR*. Helsten et al, identified the *FGFR2/FGFR3* and *TAAC3* fusion as one with high frequency.^96^ In triple negative breast cancer (TNBC), *FGFR2* fusion partners *AFF3, CASP7,* and *CCDC6* aberrantly activate the gene, while in lung cancer other two fusions were detected (*FGFR3-TACC3; FGFR2-CIT*). Fusion between *FGFR3* and *TACC3* was also identified in glioblastoma, cervical SC and urothelial carcinoma. Type I FGFR fusions were detected in patients with AML, acute lymphoblastic leukemia (ALL) and peripheral T cell lymphoma (PTCL).^109^ Therefore, cells harboring these FGFR fusions develop oncogenic properties.

The most frequent genomic alteration of the FGFRs is gene amplification, *FGFR1 and FGFR4* having the highest frequencies. *FGFR1* amplification is common in HR+ cancers, HER2+ cancers and TNBC, and is associated with poor prognosis.^99^

Rarely, mutations can occur in residues that are not present in common isoforms, as observed in most cancers. For example, in head and neck squamous cell carcinoma (SCC), a rare mutation, P11362-21 G33R, was detected in an uncommon isoform of FGFR1, while mutations in the common isoforms were not found.^110^ In the case of FGFR2, mutations in non-common isoforms were identified in various cancers: bladder cancer (P21802-20 M71T), CRC (P21802-20 R88H, R95Q, D221N), lymphoma (P21802-20 M71T), and lung adenocarcinoma (p.R496T).^106,111,112^ FGFR3 presented a unique mutation in an isoform different from the common ones, specifically P22607-4 P688S in BC.^112^ For FGFR4, no mutations were detected in isoforms other than the common ones.^112^

A synthesis of FGFR receptors is presented in Table 3, providing details regarding the genes that encode FGFR proteins, mutations that may be or not be oncogenic and the disease where the FGFR mutations occur. Moreover, different mechanisms of action are presented in the below table, highlighting how diverse the FGFR activity might be in different human oncological diseases.

**Table 3:** Overview of FGFR mutations and their role in cancer progression

Receptor	Gene family/symbol	Disease	Mechanism	Mutation	Oncogenic	Reference
FGFR	*FGFR1*	Glioneuronal tumors	Alters autophosphorylation	N546K	Yes	^843,844^
FGFR	*FGFR1*	Esophageal adenocarcinoma Glioneuronal tumors	Increased kinase activity	K656E	Yes	^843–846^
FGFR	*FGFR1*	Gallbladder and breast cancer (BC)	Unknown	S125L	Unknown	^102,103^
FGFR	*FGFR1*	Colorectal cancer (CRC), GC, Skin, eyes and mouth disease, BC	Point mutation in extracellular or transmembrane domain	S125L, P105S, A268S, Y374C	Yes	^842^
FGFR	*FGFR2*	NSCLC, GC and endometrial cancer	Increased receptor–ligand binding affinity	Y375C	Yes	^96,98^
FGFR	*FGFR2*	NSCLC, GC and endometrial cancer	Increased receptor–ligand binding affinity	C382Y	Yes	^96,98^
FGFR	*FGFR2*	NSCLC, GC and endometrial cancer	Increased receptor–ligand binding affinity	S252W	Yes	^96,103^
FGFR	*FGFR2*	NSCLC, GC and endometrial cancer	Increased receptor–ligand binding affinity	W290C	Yes	^96,847^
FGFR	*FGFR2*	NSCLC, GC and endometrial cancer	Increased receptor–ligand binding affinity	P253R	Yes	^96,98^
FGFR	*FGFR2*	NSCLC, GC and endometrial cancer	Unknown	N549H	Likely	^96,848^
FGFR	*FGFR2*	NSCLC, GC and endometrial cancer	Increased receptor–ligand binding affinity	K659E	Unknown	^96,849^
FGFR	*FGFR2*	Bladder cancer	Unknown	P21802-20 M71T	Unknown	^104^
FGFR	*FGFR3*	Urothelial carcinomas	Increased receptor–ligand binding affinity	R248C	Unknown	^104,105,850^
FGFR	*FGFR3*	Urothelial carcinomas	Unknown	S249C	Yes	^104,105,851^
FGFR	*FGFR3*	Urothelial carcinomas	Unknown	G370C	Yes	^104,105,848^
FGFR	*FGFR3*	Urothelial carcinomas	Unknown	Y373C	Yes	^104,105,848^
FGFR	*FGFR3*	Oral SCCs	Unknown	G697C	Unlikely	^108,852^
FGFR	*FGFR4*	RMS	Constitutively activates the receptor in ligand-dependent manner	V550E	Yes	^106,107,853^
FGFR	*FGFR4*	RMS	Unknown	N535K	Yes	^106,107,853^
FGFR	*FGFR4*	BC	Activating mutation	Y367C	Yes	^106,107,854^

### Deregulation of IR and IGF1R in cancer

Insulin receptors (IR) and insulin-like growth factor 1 receptor (IGF1R) regulate metabolic processes, particularly glucose homeostasis, through modulation of PI3K/Akt and Ras/MAPK pathways, impacting metabolism, cell growth, differentiation, and survival, with implications extending to cancer research.^62^

Insulin and IGF-1 influence biological mechanisms via the IR and IGF1R. IR and IGFR1 are members of the insulin receptor family, among orphan insulin receptor-related receptor and are responsible for the maintenance of glucose homeostasis, as well as glucose uptake and its conversion into fat, thus modulating the insulin secretion and other metabolic processes.^113^

IR and IGFR1 play crucial roles in cancer procession and development, overactivation of these receptors is common is cancer cells, with a particular overexpression in dedifferentiated cell, leading to resistance to different anti-tumor therapies.^114^ Strong evidence suggests the link between type 2 diabetes mellitus, obesity and the development and progression of tumors,^115–117^ thus, even if the IR pathway gained attention for the antidiabetic therapies, nowadays it represents a target for antitumor therapies.

IRs, upon ligand binding, undergo autophosphorylation, activating growth factor receptor-bound protein 2 (GRB2) and the p85 subunit of PI3K. This leads to Akt activation, regulating metabolic enzymes and influencing cell growth, proliferation, and survival, critical processes in tumor development.^118^

Multiple studies have demonstrated the implication of insulin receptor (IR) pathway in cancer development and progression. Aberrant overactivation of IR pathway is common in cancer cells, mostly in stem-like cells and could be related to drug resistance. Insulin and Insulin-like growth factors I and II bind to IR and IGF-IR, two receptors with high structural similarities that are responsible for glucose metabolism, cell growth and proliferation. As presented in Table 4, in cancer, this pathway is altered and may serve as targets for cancer therapy.^119–122^

**Table 4:** Overview of insulin receptors mutations and their role in cancer progression

Receptor	Gene family/symbol	Disease	Mechanism	Mutation	Oncogenic	Reference
IGF-IR	Unknown	SCC	Partial disruption of normal IGF1R activity regulation	A1347V	Yes	^128^
IGF-IR	Unknown	RCC	Partial disruption of normal IGF1R activity regulation	S1278	Yes	^128^
IGF-IR	Unknown	NSCLC	Partial disruption of normal IGF1R activity regulation	M1255I	Yes	^128^
IRS1	*IRS1*	NSCLC	Gain of function; induce cell proliferation; inhibit cell migration	p.S668T p.D674H	Yes	^855^
INSR	*INSR*	Endometrial cancer	Loss of function, nonsense mutation leads to decreased insulin receptor and AKT activity	W1202X (nonsense mutation in exon 20)	Yes	^856^
IRS-1	*IRS-1*	BC	Unknown	p.Arg267Cys	Yes	^855^
IRS-2	*IRS-2*	CRC	Alteration of insulin/IGF signaling affecting cell growth and metabolic processes	p.Pro559Leu	Yes	^857^
IRS-1	*IRS-1*	CRC	Aberrant activation/inhibition of insulin/IGF pathway influencing cell proliferation	p.Gln655His	Yes	^857^
IRS-1	*IRS-1*	CRC	Disruption of normal signaling and potential impact on metabolic regulation	p.Asp1014Gly	Yes	^857^
IRS-1	*IRS-1*	RCR	Potential alteration in cellular signaling pathways and metabolic processes	p.Asp1181His	Yes	^857^
IRS-1	*IRS-1*	CRC	Possible influence on cell growth and proliferation via altered signaling	p.Pro1203Ser	Yes	^857^
IRS-2	*IRS-2*	CRC	May affect insulin/IGF signaling and downstream cellular processes	p.Asp782Asn	Yes	^857^
IRS-2	*IRS-2*	CRC	Potential modulation of IRS-2 functions relevant to tumorigenesis	p.Gly1230Ser	Yes	^857^
IR	*INSR*	RMS	Alternative splicing under hypoxic conditions, promoting the IR-A isoform over IR-B	Increased expression of IR-A isoform; alternative splicing	Yes	^858^
INSR	*INSR*	Thyroid cancer	Gain (overexpression of IR-A)	Unknown	Yes	^859^
IR	*IR gene on chromosome 19*	Cancer, type 2 diabetes	Loss and gain (depending on splicing)	Splicing enhancers and inhibitors in intron 10 and exon 11	Possible via IR-A	^860^

According to Ullrich et al., due to the high degree of similarity between IR and IGF-IR, hybrid receptors (HRs) can form when an IR alpha-beta hemi receptor combines with an IGF-IR alpha-beta hemi receptor.^123^ These hybrid receptors are expressed in all tissues along with IR and IGF-IR. The three possible receptors bind the same ligands—insulin, IGF-1, and IGF-2, with different affinities. When ligands bind to the receptors, the receptors become autophosphorylated on their TYR residues and activated intracellular signaling pathways. According to Hers et al., the downstream signaling activates the PI3K and regulates Akt via PDK1, mediating metabolic effects, cell growth, proliferation, and cell survival .^118^ In adult tissues, IR is responsible for the metabolic functions and IGF-IR are mainly regulators of growth processes.^124^ Both receptors can overlap in their biological effects in cancer cells, thus latest therapeutical concepts maintain that targeting both IR and IGF-IR would be a better approach than targeting IGF-IR alone.^122,125–127^

In the IGF1R, three cancer associated mutations were described by Craddock and Miller, two of them in the C-terminus (DS1278 and A1347V) and one in the C-terminal lobe of its catalytic domain (M1255I), mutations that disrupt the downstream signaling cascade.^128^

Table 4 provides a synthetic presentation of Insulin receptors and the oncological diseases in which they are involved, with details regarding the genes that encode the proteins, and different mutations that may act as oncogenic mutations and a description of the various mechanisms that can be disturbed by the mutations in Insulin Receptors.

### Deregulation of PDGFRs in cancer

PDGFRs, responding to platelet-derived growth factors, are involved in regulating cell proliferation and migration. They activate pathways like Ras/MAPK, PI3K/Akt, and PLCγ, influencing cell growth, angiogenesis, and wound healing. Dysregulation of PDGFR signaling is implicated in various pathologies, including cancers and fibrotic disorders.^129,130^ The delicate balance maintained by PDGFR can be disrupted by changes in the receptor or its ligands, or by the crosstalk between the pathways. Dysregulation of PDGFR signaling implies a wide spectrum of disorders, even cancers. In oncological disorders, aberrant PDGFR activation can fuel uncontrolled proliferation and migration. The dysregulation of PDGFR signaling underlies multiple pathological conditions, underscoring the therapeutical potential of the regimens in oncological pathologies and not limited to them.^131–133^

The PDGFR family consists of PDGF alpha, PDGF beta, SCF receptor (Kit), CSF-1 receptor (Fms) and Flt3. PDGF alpha and beta bind homodimers of PDGF-A/B/C and D polypeptides and the heterodimer PDGF-AB. CSF-I bind the IL-34 and CSF-1, while SCF and Flt3 receptors bind one ligand each. All ligands for PDGFR family are dimeric molecules.^134^

c-KIT, essential for hematopoietic stem cells, melanocytes, and germ cells, activates multiple pathways, including Ras/MAPK, PI3K/Akt, and PLCγ. Its role in cell survival and proliferation, particularly in hematopoietic and melanogenic cells, makes it significant in various cancers. c-KIT mutations are targeted by specific kinase inhibitors in cancer therapy.^135,136^

PDGFR alpha mutations occur within the autoinhibitory juxta-membrane region (exon 12 mutations) and the kinase domain (exons 14 and 18). V561D mutation occurs in exon 12, while D842V and D842Y in exon 18. All these mutations disrupt the signaling and act as oncogenic mutations in gastrointestinal stromal tumors (GIST).^137^ Among all the PDGFRA mutations previously discussed, D842V is one of the most widely investigated and clinically significant mutations.^138^ It results in a gain-of-function in PDGFRA, which enables constitutive kinase activation without the need for ligand binding.^139^ This ongoing activation stimulates downstream signaling pathways that support cell survival and proliferation, including PI3K/Akt/mTOR and MAPK.^140^ Moreover, it is linked to a specific subset of GISTs and is present in around 5–6% of these tumors.^138^ It has been reported that this mutation is susceptible to crenolanib but resistant to certain kinase inhibitors, such as SU11248.^139,140^ Furthermore, in contrast to other PDGFRA mutations in GISTs, PDGFRA D842V has been connected to certain clinicopathological characteristics.^138^

V561D mutation occurs in the juxta-membrane domain and is another noteworthy mutation that has oncological implications. The regulatory function of the kinase can be disrupted, resulting in the incorrect activation of the enzyme and consequently the initiation of signaling pathways that lead to cell growth and survival. In contrast to the D842V mutation, the V561D mutation in PDGFRA may still exhibit sensitivity to specific tyrosine kinase inhibitors (TKIs).^140,141^ Patients with this mutation may exhibit therapeutic responses, as they could potentially respond well to TKI treatment.^142^ However, apart from GIST, the occurrence of activating c-KIT and PDGFR mutations in other types of human malignancies is extremely uncommon. Hence patients afflicted with these malignancies, although exhibiting an excessive amount of c-KIT and/or PDGFR, are unlikely to derive any advantages from imatinib-targeted therapy.^143^

PDGFR Gain-of-function mutations are identified in PDGFRA in several diseases such as Y266C, Ins450C, Del (8,9), V536E, Ins544V, N659X, D842X, and Ins491A in glioblastoma; D842X, N659X, and V561D in GIST. In PDGFRB, gain-of-function mutations were identified in unicentric Castleman disease (N666X) and multiple mutations in non-oncologic diseases.^144^

In patients with pediatric glioma, D842V, N659K, E229K, C235R, Y288C, and C290R were identified as missense mutations, E7del, E10del2, E10del as deletions and C450ins, A491ins and V544ins as insertions. Some oncogenic mutations can confer resistance to small molecule inhibitors; thus, the therapeutic approaches may need improvement.^145^

In AML, there are two groups of mutations that activate the FMS-like tyrosine kinase-3 (FLT3) gene. These mutations are known as FLT3-internal tandem duplications (FLT3-ITDs) and FLT3 point mutations in the tyrosine-kinase domain (FLT3-TKD). FLT3-ITDs occur in the juxta-membrane (JM) domain and are present in up to 25% of patients with AML. FLT3-TKD mutations, on the other hand, occur in the tyrosine-kinase domain and are found in up to 10% of AML patients. However, a novel category of activating point mutations (PMs) has been discovered, which are concentrated in a 16-amino acid segment of the FLT3 juxta-membrane domain (FLT3-JM-PMs).^146^

PDGFR mutations have a significant impact on the protein function. Multiple mutations are detected in the genes that encode PGDFR proteins and most of them seem to be oncogenic and produce imbalance in the normal physiological function of the receptor. The details related to the mechanism of action and the oncological disorders in which PDGFRs are involved are detailed in Table 5.

**Table 5:** Overview of PDGFR mutations and their role in cancer progression

Receptor	Gene family/symbol	Disease	Mechanism	Mutation	Oncogenic	Reference
PDGFRA	*PDGFRA*	GIST	Gain of function Enables constitutive kinase activation without the need for ligand binding which stimulates downstream signaling pathways that support cell survival and proliferation. Provides primary resistance to imatinib and sunitinib due to conformational change in the kinase domain.	D842V	Yes	^861–868^
			Ligand-independent kinase activation	Del DIMH842-845	Yes	^869^
			Ligand-independent kinase activation	Del HDSN845-848P	Yes	^869^
PDGFRA	*PDGFRA*	GIST	Gain of function Enables constitutive kinase activation without the need for ligand binding which stimulates downstream signaling pathways that support cell survival and proliferation. May still exhibit sensitivity to specific tyrosine kinase inhibitors (TKIs).	V561D	Yes	^140,141,870,871^
			Ligand-independent kinase activation	Ins ER561-562	Yes	^869^
			Ligand-independent kinase activation	Del RVIES560-564	Yes	^869^
			Ligand-independent kinase activation	Del SPDGHE566-571R	Yes	^869^
PDGFRA	*PDGFRA*	GBM	Gain of function V536E Promotes cell proliferation by activating signaling pathways including ERK and STAT5, even in the absence of a ligand. The mutation affects the packing of the helices in the transmembrane domain of the receptor dimer. PDGFRA^Δ^^8,9^ ligand-independent receptor activation	V536E PDGFRA^Δ^^8,9^	Yes	^872,873^
PDGFRA	*PDGFRA*	Melanoma	Gain of function: P577S, G853D—sensitive to crenolanib and imatinib	Exon 12: V561A D568N P577S Q579R Exon 14: Q639stop A663V Exon 18: K830R I834V Y849C G853D	Unknown	^874^
PDGFRA	*PDGFRA*	Melanoma	Gain of function: V658A—sensitive to crenolanib and imatinib-resistant R841K—sensitive to crenolanib and imatinib	Exon 12: S584L Exon 14: V658A Exon 18: H816Y L839P R841K	Unknown	^874^
PDGFRA	*PDGFRA*	Melanoma	Gain of function: H845Y—sensitive to crenolanib and imatinib	Exon 18: H845Y	Unknown	^874^
PDGFRA	*PDGFRA*	Melanoma	Gain of function	Exon 14: A633T K646E	Unknown	^874^
PDGFRA	*PDGFRA*	Pediatric HGG	ligand-independent activation of the PI3K pathway, promoting cell proliferation sensitive to small molecule inhibitors	Missense mutations: Y288C, D842V, N659K, N659K, E229K, C235R, C290R In-frame deletions/insertions: E7del, E10del2, E10del, C450ins, V544ins, A491ins Gene fusion: KDR-PDGFRA	Unknown	^145^
PDGFRB	*PDGFRβ*	Familial Infantile Myofibromatosis	Deregulation of PDGF signaling (p.Arg561Cys - Weakens the autoinhibitory function of the JM domain that normally prevents receptor activation under normal conditions; p.Asn666Lys—Possibly results in a structure that closely resembles the active state of KIT kinase)	p.Arg561Cys p.Asn666Lys	Unknown	^875,876^
KIT	*c-KIT*	GIST	Gain of function: ligand-independent receptor activation promoting proliferation and inhibiting apoptosis (Ras/Raf/MAPK, JAK/STAT3 and PI3K/Akt/mTOR activation)	p.W557_K558 deletion KITdelinc557/558 Intron 10/exon 11 junction deletions (resulting in p.K550_K558 deletion) Single nucleotide substitutions Duplications Homo/hemizygous *KIT* exon 11 mutant	Unknown	^877–881^
KIT	*c-KIT*	Leukemia	Gain of function: Overexpression—promotes proliferation, differentiation, and activation of hematopoietic progenitor cells.	Val560Gly sp816Val	Unknown	^882^
KIT	*c-KIT*	Melanoma	Gain of function resulting in activation of the downstream MAPK and PI3K/AKT signaling pathways	L576P K642E	Unknown	^135,883,884^
KIT	*c-KIT*	BC	Gain of function: Overexpression—supports cell survival and proliferation.	p.M541L	Unknown	^885^
KIT	*c-KIT*	Mastocytosis	Gain of function: Overexpression of c-kit D816V—leads to imatinib resistance	Missense mutations: D816V, D820G, N822I/K, F522C, V560G/I Deletions:ccodon p.A502_Y503dup, Codon 419	Unknown	^886–888^
KIT	*c-KIT*	Germ Cell Tumors	Gain of function: Activating mutations in exon 17 Overexpression of c-kit	D816V D816A D816H D820V L576P Y823C N822K Δ57 bp (codon555-573)	Unknown	^888–890^
CSF-1R	*CSF-1R*	Myelodysplastic syndrome/AML	L301S—Ligand-independent activation Y969F—Involved in negative regulatory activity	L301S + A374X Y969F	Unknown	^891,892^
CSF-1R	*CSF-1R*	RCC	Ligand independence and constitutive activation of the RTK	c.908 T > C	Unknown	^893^
FLT3	*FLT3*	AML	Ligand independent activation through dimerization and transphosphorylation	FLT3-internal tandem duplications (FLT3-ITDs); Point mutations in the tyrosine-kinase domain (FLT3-TKDs): G831; R834; D835; I836; Δ836; D839; S840; N841; Y842; Point mutations in the juxta-membrane domain (FLT3-JM-PMs): Y572C; F590GY591D; T591; V592A; F594L; V579A; Y591C;	Unknown	^146,894^

### Deregulation of VEGFRs in cancer

Vascular endothelial growth factor receptors (VEGFR-1, -2, and -3) play crucial roles in angiogenesis and vascular permeability by binding vascular endothelial growth factor (VEGF). Activation of the Ras/MAPK, PI3K/Akt, and PLCγ pathways influences wound healing, angiogenesis and vascular development. Targeting VEGFRs is critical in anti-cancer therapies, especially in inhibiting tumor blood supply due to their involvement in pathological angiogenesis.^147^

Among its ligands, VEGFR plays critical roles in physiological and pathological angiogenesis, being a key target in cancer. VEGFR family includes receptors which have different roles: VEGFR-1 (Flt-1), VEGFR-2 (KDR/Flk-1) and VEGFR-3 (Flt-4), the first two with roles in angiogenesis and Flt-4 in lymphangiogenesis.^148,149^ Furthermore, VEGFRs pathways are connected by a crosstalk with other pathways involved in cell survival, cell migration, actin reorganization, focal adhesion and proliferation, thus any structural and functional changes in the receptors may lead to imbalance in many other biological processes.^150^

Mokhdomi et al. investigated mutational patterns in 10 exons of VEGFR-1, identifying 10 genotypic variations with distinct allelic frequencies, including 8 novel variants and 2 known ones. Notably, analysis of the global SNP database unveiled the rs730882263:C>G mutation in VEGFR-1, resulting in the VEGFR-1 p.Cys1110Ser variant within the catalytic domain. This mutation potentially contributes to colon cancer pathogenesis.^151^ Moreover, VEGFR-1 rs7993418 polymorphism was associated with hematogenous metastases in GC.^152^

Two frequent gain-of-function mutations in VEGFR-2, R1051Q and D1052N were related to an increased enzymatic activity of the receptor. R1051Q variant stimulates PI3K/Akt signaling in tumor cells leading to resistance to therapy.^153^ In melanoma, using SK-MEL-31 cells as a model, R1051Q mutation activated the receptor, stimulating melanoma progression without ligand-binding.^154^ In the absence of a ligand, VEGFR-2 can form phosphorylated dimers. In this case, conformational switch of extracellular, intracellular, and transmembrane domains of the receptor are of major importance. Engineered transmembrane domain mutations such as E764I-T771I-F778I and N762I-V769I-G770I, have a crucial role in VEGRF-2 dimer stabilization by affecting its phosphorylation status.^155^ On the other hand, C482R pathogenic mutation leads to an increase in phosphorylation even in the absence of ligands. This mutation is linked to infantile hemangioma.^156^

VEGFR-2 high expression and single nucleotide polymorphisms rs1870377 A>T and rs7692791 were correlated with GC prognosis and poor survival.^157,158^ In the case of CRC, VEGFR-2 1192C/T and −604T/C single nucleotide polymorphisms are associated with microvessel density in tumor tissue.^159^ Other VEGFR-2 alterations might be correlated with Alzheimer’s disease based on a study performed on plasma samples obtained from mild cognitive impairment and Alzheimer’s disease patients.^160^

VEGFR-3 expression is correlated with tumor progression by means of lymphatic metastasis (in the case of breast, lung, ovarian, renal cell, colorectal, gastric, oral, cervical, prostate, pancreatic cancer and basal cell carcinoma) or angiogenesis (in the case of ovarian, colorectal, gastric, cervical, prostate, pancreatic, melanoma, laryngeal cancer).^161^ On the other hand, VEGFR-3 missense mutations are associated with different forms of autosomal dominant primary lymphedema,^162^ for example Milroy disease.^163^

Table 6 depicts the importance of VEGFR in the development of several malignancies and highlights the genes that encode the proteins and specific mutations that occur within these genes leading to an overstimulated receptor, or an unfunctional protein that creates the proper conditions for malignant cells to develop and proliferate.

**Table 6:** Overview of VEGFR mutations and their role in cancer progression

Receptor	Gene family/symbol	Disease	Mechanism	Mutation	Oncogenic	Reference
VEGFR-1	*FLT-1*	Angiosarcoma	Unknown	c.542G>A	Unknown	^895^
VEGFR-1	*FLT-1*	GC	Unknown	rs7993418	Oncogenic	^152^
VEGFR-1	*FLT-1*	CRC	Allosteric activation	rs730882263:C>G	Unknown	^151^
VEGFR-2	*KDR*	Hemangioma	Amplification of VEGF/VEGFR-2 signaling = gain of function	C482R	Increase angiogenesis	^156,445^
VEGFR-2	*KDR*	Melanoma, BC	Gain of function	R1051Q	Pro-oncogenic	^153,154^
VEGFR-2	*KDR*	Melanoma, BC	Gain of function	D1052N	Pro-oncogenic	^153^
VEGFR-2	*KDR*	CRC	Reduced function	L840F	Resistance to VEGFR-2 inhibitors	^896^
VEGFR-2	*KDR*	CRC	Gain of function	R961W	Unknown significance	^897^
VEGFR-2	*KDR*	CRC, melanoma	Loss of function	R1032Q	Oncogenic, increasing sensitivity to VEGFR-2 inhibitors	^154,896,898^
VEGFR-2	*KDR*	CRC, melanoma	Loss of function	S1100F	Oncogenic	^154,896,898^
VEGFR-2	*KDR*	CRC, BC	Gain of function	D717V	Oncogenic	^896,899^
VEGFR-2	*KDR*	CRC	Gain of function	G800D/R	Oncogenic	^896^
VEGFR-2	*KDR*	CRC	Gain of function	G843D	Oncogenic	^896^
VEGFR-2	*KDR*	CRC	Gain of function	S925F	Oncogenic	^896^
VEGFR-2	*KDR*	CRC	Gain of function	R1022Q	Oncogenic	^896^
VEGFR-2	*KDR*	BC	Gain of function	A1065T	Oncogenic	^899^
VEGFR-2	*KDR*	Temporal bone SCC	Unknown	p.Gln472His, c.1416A>T	Unknown	^900^
VEGFR-2	*KDR*	GC	Unknown	rs1870377 A>T	Unknown	^157^
VEGFR-3	*FLT 4*	angiosarcoma	Unknown	p.G1276E	Metastatic	^895^
VEGFR-3	*FLT 4*	angiosarcoma	Unknown	R1070L	Unknown	^895,901^

### Deregulation of HGFRs in cancer

The primary HGFR, c-Met, is involved in cell motility, invasion, and metastasis. Its activation primarily leads to the stimulation of the Ras/MAPK, PI3K/Akt, and STAT pathways. Dysregulation of c-Met is linked to various cancers, making it a target for therapies aimed at inhibiting metastatic spread.^164^

c-Met, known as hepatocyte growth factor receptor, orchestrates cell motility, invasion, and metastasis by activating signaling pathways such as Ras/MAPK, PI3K/Akt, and JAK/STAT, aberrant activation of which is linked to diverse cancers, notably driving invasive and metastatic growth, rendering c-Met a prominent therapeutic target, with multiple inhibitors devised to counter its oncogenic activity in cancer therapy.^165^

c-MET and RON have similar biochemical properties and share similar structures. C-MET is recognized by HGF while RON has its specific ligand, the macrophage-stimulating protein. RON distribution is restricted to cells that have epithelial origin and studies have demonstrated that RON expression is required for attenuating the inflammatory response, controlling the macrophages activities during infections.^166^ RON overexpression was observed in cancers localized in pancreas, bladder, lung, breast, colon, thyroid and skin, its overexpression is correlated with advanced clinical stages, and it seems that RON can modulate cell growth and migration via MAPK/Akt pathways sustaining tumorigenicity.^167–170^

Hepatocyte growth factor receptor protein is a single pass tyrosine kinase receptor which is key in embryogenesis and wound healing. Abnormal activation of *MET* in different cancers correlates with poor prognosis, enhanced angiogenesis and Epithelial to Mesenchymal Transition (EMT).^171–176^

The c-Met pathway is a potential target therapy in cancers.^172^ The activation or hyperresponsiveness of the HGFR/HGF pathway involves two primary mechanisms: mutations in MET within the extracellular or cytoplasmatic domain, resulting in prolonged biochemical signaling, and ligand-independent activation, characterized by the overexpression of the wild-type protein. The two mechanisms can act individually or concomitantly. Multiple point mutations were identified in the semaphoring, immunoglobulin plexin transcription, juxta-membrane (JM) or tyrosine kinase (TK) domains of MET, as Sattler and Reddy stated in their work.^172^ N375S mutation was identified in NSCLC, small cell lung cancer (SCLC), mesothelioma, and melanoma^177–180^; T992I in NSCLC, SCLC, mesothelioma and BCs and other mutations specifically identified in other cancer subtypes.^181–184^

Although there are limitations on the activation of cMET induced by HGF, dysregulated signaling of HGF-cMET has been detected in several malignant neoplasms.^185^ Abnormal cMET activation is possible through processes that are not dependent on HGF, such as MET mutations, gene amplification, and transcriptional upregulation.^172^

Jeffers and his group generated several fibroblast cell mutants (NIH 3T3 cells) that were inoculated in mice models, and compared to the wild type, M1258T; Y1248H; D1248H; D1246N; Y1248C; V1238I; V1206L, and M1149T clones induced tumor growth in mice.^186^ These mutations were identified with high frequency in patients with RCC.^187–190^ Furthermore, several in vivo studies have demonstrated that the activation of the HGF–cMET signaling pathway is a critical factor in promoting cancer invasion and metastasis.^191,192^

Elevated levels of HGF in both tumor tissues and plasma have been observed in patients with various types of cancers, such as invasive breast carcinoma, glioma, and multiple myeloma.^193–195^ The information regarding mutational status of these receptors and the mechanism of action in different malignancies are detailed in Table 7, with specific details regarding the encoding genes and the oncogenic status of the mutations that were detected in each receptor.

**Table 7:** Overview of HGFR mutations and their role in cancer progression

Receptor	Gene family/symbol	Disease	Mechanism	Mutation	Oncogenic	Reference
HGFR	*MET*	NSCLC, SCLC, mesothelioma, RCC	Gain of function Abnormal MET activation; mutations in MET; ligand-independent activation	N375S, T992I, M1258T, Y1248H, D1248H, D1246N, Y1248C, V1238I, V1206L, M1149T	Yes	^171–176,186–190^
HGFR	*MET*	GBM	HGF/MET signaling in glioblastoma disrupts cell cycle, proliferation, and apoptosis, and influences angiogenesis and EMT. It involves RTK activation and PI3K/Akt and MAPK pathway modulation	MET/HGF mutations and overexpression; MET amplification and overexpression	Yes	^902^
HGFR	*MET*	NSCLC, brain glioma	GF/MET signaling contributes to invasion, metastasis, and drug resistance. MET-Dexon14 mutation and MET JM domain deletion impact responses to MET inhibitors and influence the tumor microenvironment.	MET-Dexon14 mutation	Yes	^903^
HGFR	*MET*	NSCLC	Gain of function HGF-MET signaling leads to intrinsic and acquired resistance to EGFR-TKIs, involving HGF expression, T790M mutation, and MET amplification, affecting MAPK-ERK1/2 and PI3K-Akt pathways	HGF expression, T790M secondary mutation, MET amplification	Yes	^904^
HGFR	*MET*	NSCLC, gastric, prostate, RCC	Gain of function	MET or HGF overexpression, amplification, or mutation	Yes	^905^
HGFR	*MET*	BC, endometrial, hepatocellular, RCC, gastric, CRC, bladder cancers, SCLC, melanoma, HPRC, childhood hepatocellular carcinoma, head and neck SCCs	Gain of function; Dysregulation leading to tumor growth, invasion, metastasis	T1010I, R988C, R970C, T992I, N930S, P991S, N375S, M431V, N454I	Yes	^906^
HGFR	*MET*	NSCLC	Gain of function Resistance to second-generation EGFR-TKIs	T790M	Yes	^907^

### Deregulation of MuSK

Muscle-specific Kinase (MuSK) is crucial in neuromuscular junction formation. Its activation stimulates pathways like PI3K/Akt and MAPK, which are essential for the clustering of acetylcholine receptors and development of the postsynaptic membrane. MuSK’s role in muscle function makes it a focus in neuromuscular disorder studies.^196,197^

MuSK is a RTK that is required for the maintenance and formation of the neuromuscular junction and its ligand is agrin, which triggers the signaling cascade via casein kinase 2 (CK2), Dok-7 and rapsyn.^198–201^ The activation of MuSK requires also its coreceptor LRP4 and a stoichiometric ratio of 1:1:1 agrin: LRP4: MuSK complex being essential for its function.^202^With around 100 kD and a single-pass transmembrane RTK, Musk interacts with agrin and LRP4 to modulate the postsynaptic apparatus.^203^ Communication from the motoneuron to the muscle is essential for the formation and maintenance of the neuromuscular junction, thus Musk plays a crucial role in the acetylcholine receptor clustering during the development of the neuromuscular junction.^204^

Because of the role in neuromuscular junction maintenance, Musk is not studied in cancer related subjects, and the literature is focused on its role in autoimmune diseases such as Myasthenia gravis or other neuromuscular disorders.^205^ Although not related to oncological disorders, there is one mutation in MuSK that might be responsible for myasthenic syndrome, M835V, which induces changes in the receptor structure, therefore disturbing the downstream signaling.^206^ Other two mutations (c.2062C>T (p.Q688X) non-sense mutation and c.2324T>C (p.F775S) missense mutation) that are not recorded in Human Gene Mutation Database, were identified in a case of Chinese neonatal congenital myasthenic syndrome. The missense mutation was inherited from the mother and was predicted as pathogenic and severe by various bioinformatics programs.^207^

### Deregulation of ALK in cancer

Anaplastic lymphoma kinase (ALK) plays a role in the development of the nervous system and is implicated in various cancers. It activates pathways such as Ras/MAPK, PI3K/Akt, and JAK/STAT, contributing to its oncogenic potential. ALK is a target for cancer therapy, especially in anaplastic large cell lymphoma and NSCLC.^208–210^

In addition to its role in cancer, ALK and its receptor family member Leukocyte tyrosine kinase receptor (LTK) have a key role in the normal physiology of the central nervous system. It was observed that in the absence of LTK and ALK, neuronal migration, neural progenitor populations and cortical layers patterning were disrupted, thus some researchers suggest that the use of ALT/LTK inhibitors in cancer patients should be carefully monitored to avoid brain dysfunctions.^211^ Moreover, the mammalian RTK ALK was first described as the product of the t(2;5) chromosomal translocation found in non-Hodgkin’s lymphoma.^212,213^

The physiological roles of LTK while not fully understood, have been partially explained through some in vivo studies. It was observed that mice that had ALK/LTK knockout genes had significant reduction in newborn neurons, suggesting that ALK may play an important role in the generation or survival of these neurons, during neurodevelopment.^214,215^ The status of LTK and ALK as “orphan” receptors changed with the identification of their ligands, ALKAL1 and ALKAL2.^216–218^

Roll and Reuther evaluated the ALK activating mutations in LTK, using a benign tumor model (pheochromocytoma—adrenal gland benign tumor) in mice.^219^ This study identified specific ALK mutations like F1147L and R1275Q, and the corresponding LTK mutations F568L and R669Q. Moreover, the F1147L and R1275Q mutations are frequently detected in neuroblastomas.^220–222^ The two mutations are responsible for the constitutive activation of ALK in neuroblastoma.^223^ ALK/LTK mutations and their role in this specific malignancy are presented in Table 8.

**Table 8:** Overview of ALK/LTK mutations and their role in cancer progression

Receptor	Gene family/symbol	Disease	Mechanism	Mutation	Oncogenic	Reference
ALK	*ALK*	Neuroblastoma	Constitutive activation of ALK	F1147L	Unknown	^222^
ALK	*ALK*	Neuroblastoma	Constitutive activation of ALK	R1275Q	Unknown	^222^
LTK	*LTK*	Neuroblastoma	Induce cellular transformation	F568L	Generate mutations	^219,222^
LTK	*LTK*	Neuroblastoma	Induce cellular transformation	R669Q	Generate mutations	^219,222^

### Deregulation of ROS1 in cancer

ROS1 belongs to the human RTKs and is the only member of the ROS1 family. *ROS1* was evaluated first in solid tumors in 1987 and it was revealed that in glioblastoma cell line U118MG, *ROS1* was altered.^224^ *ROS1* rearrangements were observed in NSCLC, initially in 2007, together with the discovery of *ALK* rearrangements in NSCLC.^225–227^ Moreover, multiple fusions were detected, most of them in less than 2–3% of these cases. However, fusions with CD74 were detected in almost half of the cases,^228^ fusions with EZR in not more than 24%,^229^ with SDC4, TPM3 and SLC34A2 in less than 15%.^228–230^ Such rearrangements were also reported in cholangiocarcinoma, in 8.7% of the samples that were analyzed.^231^ ROS1 acts like a driver in various cancers, including NSCLC.^232,233^ It activates signaling pathways like Ras/MAPK, PI3K/Akt, and JAK/STAT, which contribute to its role in oncogenesis. ROS1 has become an important focus in cancer therapy, with targeted inhibitors showing effectiveness against ROS1-driven cancers.^234^

Point mutations in ROS1 such as D2033N, S1986F, L2000V, G2032K, G2032R and L2086F, have been associated with resistance to therapy. These mutations are linked to a poor overall survival rate, as detailed in Table 9 which highlights the mechanism of action of each detected mutation, even if their oncogenic status is not known yet.^235–238^

**Table 9:** Overview of ROS1 mutations and their role in cancer progression

Receptor	Gene family/symbol	Disease	Mechanism	Mutation	Oncogenic	Reference
ROS-1	*ROS1*	NSCLC	Induce resistance to Lorlatinib	G2032R	Unknown	^908,909^
ROS-1	*ROS1*	NSCLC	Induce resistance to Lorlatinib	G2032K	Unknown	^908^
ROS-1	*ROS1*	NSCLC	Aquired Resistance to crizotinib	D2033N	Unknown	^909,910^
ROS-1	*ROS1*	NSCLC	Resistance to crizotinib	S1986F	Unknown	^909,910^
ROS-1	*ROS1*	NSCLC	Induce resistance to Lorlatinib	L2000V	Unknown	^235^
ROS-1	*ROS1*	NSCLC	Induce resistance to Lorlatinib	G2032K	Isolated case	^235^
ROS-1	*ROS1*	NSCLC	Induce resistance to Lorlatinib	L2086F	Unknown	^235^

ROS1expression is undetectable in most normal tissues, expressed at low levels in parathyroid glands, eyes, skeletal muscle, larynx and adrenal glands. However, high expression levels were found in the cerebellum, peripheric nerves, colon, kidney and stomach.^239–241^

### Deregulation of RET in cancer

Rearrange during transfection (RET) is the RTK that is interacting with ligands at the cell surface and plays a key role in the development of the central and peripheral nervous system.^242^ RET is crucial for the development of the enteric nervous system and kidneys. It primarily activates the Ras/MAPK, PI3K/Akt, and PLCγ pathways. Mutations in RET are associated with multiple endocrine neoplasia^243^ and Hirschsprung’s disease,^244^ making it a significant focus in developmental biology and genetic disease research.^245^

RET was discovered as a protooncogene; thus, a large body of research was conducted to evaluate the role and the effects of RET and its mutated forms. It seems that RET may play a key role in parathyroid hyperplasia, thyroid cancer, and lung cancer.^246^ RET fusions were found in around 20% of papillary thyroid carcinomas and 2% of the NSCLC.^245,247,248^

RET is a receptor tyrosine kinase embedded in the cell membrane, encoded by the proto-oncogene RET.^249^ RET has been recognized as playing vital roles in various developmental processes, especially in the formation of the kidney and the enteric nervous system during embryonic development.^250–252^ Changes in RET have been linked to several diseases, including Hirschsprung’s disease and various types of cancer.^253,254^ Over the past thirty years, numerous alterations in RET have been identified that lead to continuous activation of its kinase activity, a key factor in many cancer subtypes.^255–257^

The phosphorylation of specific tyrosine residues on the cytoplasmic portion of the RET receptor is key to its function. This phosphorylation enables the binding of various adaptor proteins, which are essential for transmitting external signals and activating major downstream signaling pathways. These pathways include PI3K/Akt, RAS/RAF/MEK/ERK, JAK2/STAT3, and PLCγ.^258^

Specifically, RET phosphorylation at Y687 attracts SHP2 phosphatase, activating the PI3K/AKT pathway to promote cell survival.^259^ The tyrosine residues Y752 and Y928 serve as crucial sites for binding the Signal Transducer and Activator of Transcription 3 (STAT3), leading to its activation and movement into the nucleus, which is important for the transcription of STAT3 target genes.^260^ Phosphorylation at RET -Y905 maintains RET in an active state and is vital for attaching to adaptor proteins Grb7/10.^261,262^ Moreover, RET -Y981 is essential for the activation of Src kinase.^263^

PLC- γ interacts with phospho- RET at Y1015, subsequently activating the PKC pathway.^264^ The phosphorylation of RET at Y1062 is critical for recruiting adaptor proteins that trigger the activation of the PI3K/Akt, RAS/RAF/MEK/ERK, and MAPK pathways.^265^ Lastly, Grb2 binds to phospho-RET at Y1096, facilitating the activation of the RAS/RAF/MEK/ERK pathway, which is important for cell proliferation and differentiation.^266,267^

M918T mutation in RET was identified in thyroid gland carcinoma, as a gain of function mutation, increasing the substrate binding and conferring drug resistance.^268,269^ Furthermore, RET A883F mutation, also a gain of function mutation, increased RET kinase activity in solid tumors of thyroid cancer.^270–272^

Using new NGS platforms for analysis, human tumor specimens were used to discover RET mutations in other cancers such as breast carcinoma (RET C634R), endometrial and Merkel-cell carcinomas (RET E511K) and RET V804M in CRC and hepatoma.^273^ RET C634R is a gain of function mutation, resulting in an autophosphorylation of RET,^274^ RET 3511K, a gain of function mutation, increased RET and ERK phosphorylation.^275^ Another gain of function mutation in RET, V804M increased the kinase activity and is considered a gatekeeper due to lack of response to inhibitors like cabozantinib in thyroid cancer.^276^

### Deregulation of ROR in cancer

ROR1 and ROR2, involved in developmental processes and cancer progression, primarily modulate the Wnt signaling and JAK/STAT pathways. Their roles in cell migration and cancer progression, particularly in the context of Wnt signaling, make them potential targets in oncology.^277^

ROR is a small RTK family, including ROR1 and ROR2 which were first characterized in 1992 form SH-SY5Y neuroblastoma cell line.^277,278^ The two receptors are highly expressed during embryogenesis and not expressed in adult tissue; however, an increased expression of the ROR receptors is observed in tumor tissues with increased cell proliferation.^279–282^ According to Zhao et al., no mutation in ROR1 have been found yet,^283^ however the overexpression of ROR1 itself is a cause of increased proliferation and cell growth, in malignancies, ischemia and diabetes.^284–286^

In the case of ROR2, no mutation was detected in cancers, however, a study focused on 21 patients with short stature, identified 10 missense, one nonsense and one frameshift mutation. The only mutation that had a potential effect on the downstream Wnt5a-ROR2 pathway was G559S which may disturb the subcellular localization and protein expression.^287^

### Deregulation of Eph family of receptors in cancer

Erythropoietin-producing human hepatocellular Receptors (Ephs), including EphA and EphB, are involved in developmental processes in the nervous system. They primarily activate the Ras/MAPK and JAK/STAT pathways. Their implication in cancer progression and metastasis has made them a focus in cancer research.^288^

Ephs are a group of receptors that become active when binding to their ephrins; Ephs are divided into two subclasses EphA and EphB.^289^ The Ephs are activated by a cell-cell interaction with their ligands that are membrane-bound proteins, and the signaling is involved in embryogenic development, cell migration, and segmentation^290,291^ and play a key role in angiogenesis, stem cell differentiation and cancer progression.^292,293^ Eph receptors represent the biggest RTK family, with nine EphA receptors (A1-A8 and A10) and five EphB receptors (B1-B4 and B6).^294–296^

Deficiency in autophosphorylation of EphA3 was observed for D678E and R728L mutant forms. The N85S and T116N mutations in the binding domain and D219V and S229Y in sushi domain and M269I in EGF domain showed differences in the ephrin-binding ability, compared to wild type, while other mutants showed no differences. G187R mutant was detected as very important, disrupting the conformation of the binding domain. Also, G228R and W250R mutations in sushi domain had drastically disrupted the binding.^297^

Several mutations were highlighted in different regions of the EphA3, with various degrees of Loss-of-function, impairing the binding or induce structural alterations T116N, G187R, V206L, S229Y, W250R, M269I, F311L, N379K, T393K, A435S, D446Y, S449F, G518L, T660K, D678E, K761N, R728L, G766E, and T933M.^298^

Exon 17 of EPHB4 gene was sequenced in lung cancer cell lines and patient samples and several mutations were detected: in the extracellular linker region (A230V), first extracellular fibronectin III repeat (A371V, P381S), in the extracellular juxta-membrane domain (W534*, E536K), in the TK domain (G723S, A742V) and a mutation in the intracellular linker region (P881S). Except A371V, the rest were newly discovered by Ferguson et al.^299^

Chakraborty et al. detected several structural alterations in EphA3 (A749D, W790C, F152S), EphA7 (L749F), EphB1 (G685C) and in EphB4 (V748A), all mutations inducing changes in NSCLC samples.^300^ The mutations impact in NSCLC remains unclear and needs further functional analysis for each mutation.

Faoro et al. evaluated one mutation in EphA2 which caused constitutive activation of this receptor and increases invasiveness.^301^ G391R mutation was detected in H2170 cells and 2 out of 28 SCC patient samples, but not in other subtypes of lung cancer.

The mutational status of these receptors is presented in Table 10 highlighting the disease in which each mutation was detected and the mechanism of action that led to the potential oncogenic activity.

**Table 10:** Overview of Eph receptors mutations and their role in cancer progression

Receptor	Gene family/symbol	Disease	Mechanism	Mutation	Oncogenic	Reference
EphA2	*EPHA2*	NSCLC	Alter structure	W112C	Unknown	^300^
EphA2	*EPHA2*	NSCLC	Promotes EphA2 activation and sustain invasion	G391R	Unknown	^301^
EphA3	*EPHA3*	NSCLC	Unknown	D678E	Unknown	^911^
EphA3	*EPHA3*	NSCLC	Impairing the binding or alter the structure	S229Y T116N G187R V206L W250R M269I F311L N379K T393K A435S D446Y S449F G518L T660K D678E K761N R728L G766E T933M	Yes	^298^
EphA3	*EPHA3*	CRC	Alter structure	D806N	unknown	^912^
EphA3	*EPHA3*	NSCLC	Alter structure	F152S A749N W790C	unknown	^300^
EphA7	*EPHA7*	NSCLC	Major alterations in the receptor	L749F	Yes	^300^
EphB1	*EPHB1*	NSCLC	Alter structure	G685C	unknown	^300^
EphB4	*EPHB4*	NSCLC	Alter structure	V748A	unknown	^300^
EphB4	*EPHB4*	NSCLC	Alter structure	A230V A371V P381S	Likely	^299^
EphB4	*EPHB4*	NSCLC	Alter structure	A742V	Likely	^299^
EphB4	*EPHB4*	SCLC	Alter structure	W534 E536K G723S P881S	Likely	^299^

### Deregulation of RYK in cancer

RYK, a member of the receptor-like tyrosine kinase family, is involved in Wnt signaling, influencing both β-catenin-dependent (canonical) and β-catenin-independent (noncanonical) pathways. The roles of RYK have been investigated in several model organisms, such as Drosophila, zebrafish, Xenopus, and mouse models.^302–305^

The study of RYK is significant for understanding complex developmental processes and its aberrant roles in diseases, including cancer, making it a notable focus in both developmental biology and oncology.^306^ Its implication in cancer progression, particularly in relation to Wnt signaling, has led to its exploration as a potential therapeutic target.^307^ Its regulation affects various cellular processes such as cell polarity, cell migration, skeletal development, neurogenesis, and axon guidance.^306,308^ Ryk targeted deletion in mice results in inhibited growth, abnormalities in the development of the skull and skeleton, and death after birth.^305^ Experiments conducted outside of a living organism have demonstrated that RYK has the ability to attach to Wnt, Frizzled 8, and Dishevelled proteins in order to initiate β-catenin/TCF-dependent transcription.^309,310^

Moreover, RYK has a genetic interaction Van Gogh-like 2 (Vangl2) proteins. Vangl2^−/−^; Ryk^−/−^ mice show typical phenotypes associated with planar cell polarity signaling, including problems with neural tube closure, elongation of the body axis, and craniofacial development.^311,312^ Also, within the hematopoietic system, RYK has been demonstrated to possess a cell-intrinsic influence over hematopoietic stem cells (HSCs), regulating their proliferation, apoptosis, and their capacity for repopulation.^313^

In a study led by Seon-Yeong Jeong et al., RYK role in regulating Wnt signaling in bone marrow mesenchymal cells was investigated. Previous research had indicated RYK’s influence on HSC proliferation, quiescence, and apoptosis, but the study proposed a role for RYK in the stromal component of the bone marrow. Notably, downregulating RYK in mesenchymal stromal cells (MSCs) had no impact on their cell-autonomous functions, such as proliferation and differentiation. However, it did affect MSC colony-forming activity, independent of Wnt signaling.^314^

A CRISPR-Cas9 gene-targeting model confirmed the absence of Wnt response in cell-autonomous MSC functions during differentiation and suggested a Wnt-independent role for RYK in maintaining MSC colony-forming populations. Reducing RYK also attenuated Wnt3a’s stimulatory effect on MSC niche activity, particularly in enhancing HPC self-renewal. Significantly, RYK dose-dependently modulated Wnt signaling in MSCs, influencing its intensity. These findings underscore RYK’s physiological role in regulating Wnt signaling in the bone marrow MSC niche, fine-tuning HPC self-renewal, and contributing to the control of hematopoietic activity in a homeostatic manner.^314^

RYK, along with other receptors like ROR1/2, contributes to the transmission of signals initiated by Wnt ligands, specifically Wnt5a. The signals deviate from the canonical pathway and have the potential to influence cellular processes such as cell polarity, migration, and axonal growth.^315^

Moreover, in the presence of RYK, Wnt5a influences axon growth and repulsive signal guidance via Ca^2+^-dependent signaling. Wnt5a can exhibit either tumor-suppressing or oncogenic properties in various types of cancer.^316,317^

In a study by Katso et al., assessed the H-RYK overexpression’s predictive value in epithelial ovarian cancer. The study also explored the potential role of H-RYK in angiogenesis, a critical process in tumor progression and metastasis. Despite its impaired catalytic activity, H-RYK was shown to signal through the mitogen-activated protein kinase pathway. Interestingly, H-RYK overexpression did not correlate with the proliferative status of the tumor cells, suggesting its contribution to tumorigenesis might not be through inducing excessive cell proliferation. Instead, it may play a role similar to other kinase-impaired receptors in promoting cell survival, thus contributing to carcinogenesis by protecting cells from apoptosis.^318^

### Deregulation of CCK-PTK7 in cancer

CCK4/PTK7 was first described by Mossie et al., in 1995, and its functions are not completely understood.^319^ Initially named CCK-4 (colon carcinoma kinase 4), PTK7 is regulating Wnt signaling pathways and controls morphogenesis and patterning modulating cell molarity, migration and wound healing.^320–322^ Later, it was shown that PTK7 expression could be correlated with cancer development and metastasis, while mutations in PTK7 are involved in human neural tube closure defects, scoliosis or inner ear polarity defects.^323–326^

PTK7 upregulation was detected in gastric, esophageal, colorectal, lung carcinoma or BC, while a downregulation of PTK7 was related to lung SCC, ovarian cancer and melanomas.^327–330^ Other studies underlined PTK7 role as a marker for normal colon stem cells and its potential role as a marker for tumor initiating cells in NSCLC, ovarian cancer or TNBC,^331,332^ while PTK7 inhibition showed a sustained tumor regression, indicating that some anti PTK7 therapies may have a role in tumor inhibition.^333,334^ However, no mutations have been reported to be responsible for tumor development.

### Deregulation of NGFR in cancer

Nerve growth factor receptor (NGFR), also named P75 neurotrophin receptor or CD271, is a 45 kDa receptor, consisting of a single peptide and has nerve growth factor as a ligand.^335,336^

Nerve growth factor receptor is involved in signaling for tumor development and progression, with an important role in proliferation when overactivated. Wu et al. showed that NGFR is highly expressed in metastatic lung clones of TNBC cells, the overexpression led to the growth and invasion of tumor cells to distant tissues. Moreover, the study demonstrates that NGFR is highly expressed in TNBC patients compared to non-TNBC patients, and negatively correlated with overall survival of the patients.^337,338^

TRKA is the most common oncogene in the TRK family, with significant presence in human tumors, over 7%, while TRKB and TRKC are less present. The inhibition of activated TRKA is a reliable approach in tumor inhibition. Several compounds showed inhibitory effect on NTRKs and display side effects, however as Wang et al. highlighted, no reports of selective TRKA inhibitors have been published. Wang research group discovered a TRKA selective inhibitor named 32 h which had an IC_50_ of 72 nmol/L for TRKA while for TRKB and C was above 1 µmol/L and the antitumor effect was demonstrated by cutting-edge determinations such as RNA-seq which underlined the TRKA inhibition and modulation of Wnt pathways. Furthermore, the pharmacokinetic properties have been tested and generated promising results on xenograft models suggesting that TRKA selective inhibitors can represent a therapeutic approach for NTRK1 fusion positive cancers.^339^

The TRK family, including TRKA, TRKB, and TRKC, is involved in nerve growth and survival. They activate pathways like Ras/MAPK, PI3K/Akt, and PLCγ, playing roles in both neuronal and non-neuronal tissues. Their involvement in neurodegenerative diseases and cancers makes them significant in neurological research and therapy.^340^

### Deregulation of AXL in cancer

The AXL family of RTKs, also known as TAM family includes three main receptors: TYRO3, AXL, and MER. TAM receptors are overexpressed in different malignancies, such as leukemia, melanoma, gastric, colon, lung and BC, promoting cell survival.^341–345^ AXL biosynthesis is regulated by key transcription factors (AP1, Sp1/Sp3, YAP/TAZ/TEAD, HIF1α, MZF-1) and Toll-like receptor signaling in dendritic cells and macrophages, which increase AXL mRNA expression. This process is further controlled by a feedback mechanism involving other RTKs.^346–348^ AXL regulates cell proliferation, cell cycle, cell growth, and survival.^349,350^

In adults, AXL expression is usually low but is abnormally high in several cancers, including breast, chronic lymphocytic leukemia, NSCLC, pancreatic, glioblastoma, melanoma, RCC, colorectal, prostate, and esophageal cancers.^345,351^ The overexpression of AXL is correlated with various cellular processes as follows: epithelial to mesenchymal transition, angiogenesis, chemotherapy resistance and weak antitumor immune response.^345^ The regulation of AXL involves various factors, including microRNA miR-34a at the translational level and the proteolytic release of its extracellular domain, cleaved by metalloproteinases ADAM10 and ADAM17.^352^ In addition to this, HIF1α^348^ and AP-1^347^ transcription factors and methylation of CpG islands in the promotor region^353^ were reported as AXL regulators in some studies.

AXL activation by the GAS6 ligand leads to the stimulation of various downstream signaling cascades such as JAK/STAT3, PI3K/Akt, Grb2/RAS/MEK/ERK1/2, and FAK/Src/NF kappa B.^18,354^

In a study, Salian-Mehta et al. have identified three missense AXL mutations (p.L50F, p.S202C, and p.Q361P) and one intronic variant (c.586-6C>T) in Kallman syndrome and norm osmic idiopathic hypogonadotropic hypogonadism subjects.^355^ In another study were described 53 AXL, 36 MER and 25 TYRO3 mutations identified on a cohort of 509 female patients diagnosed with endometrial adenocarcinoma.^356,357^ In all these cases the main majority were missense mutations. It is important to mention that no mutations have been detected in the KW (I/L)A (I/L)ES (a.a 714–720) motif, which is a conserved domain specific to all RTKs of the TAM family.^357^

Both AXL and TYRO3 were found to be expressed in various cutaneous melanoma cell lines.^358^ TYRO3 is particularly activated by tumor secreted protein S (ProS1) resulting in the activation of multiple pathways involved in cancer cells survival such as AKT and ERK.^359,360^ The overexpression of TYRO3 has been associated with poor survival in the case of colorectal, hepatocellular and BCs.^361^ Various mutations within the kinase domain of TYRO3 such as M592I, N615K, W708fs*5, A709T, C690R have been linked to to colon,^362^ lung,^363^ melanoma,^364^ brain cancer,^364^ and acute myeloid leukemia respectively.^365^ In the cytosolic domain have been remarked the following mutations: R462Q, R514Q and G809D which were associated with melanoma,^364^ pancreatic^366^ and colon cancer.^362^ In the extracellular and transmembrane domains have been determined Q67 and H60Q in melanoma^367,368^ and E340 in lung cancer.^363^

MER, also known as RP38, c-Eyk, c-mer, and Tyro12 is considered a proto-oncogene, playing important roles in cell survival, migration and differentiation.^369^ It was found to be upregulated in leukemia,^370^ lymphoma,^371^ colorectal,^372^ gastric,^373^ and lung^374^ cancers. Specific MER variants like P802S were found in melanoma,^375^ while another range of mutations like p.T690I, p.R20S, p.I518V, p.R466K, p.S118N, p.V870I, p.A282T, p.N498S, p.R293H, p.R865W, p.E823Q variants were identified in multiple myeloma.^376^ Moreover, MER mutations are linked with around 2% of the cases with severe autosomal recessive retinal dystrophies.^377^ Over the time have been identified 79 variants including missense (33), nonsense (12), splice defects (12), small deletions (12), small insertion-deletions (2), small duplications (3), exonic (2) and gross (3) deletions.^377^

### Deregulation of TIE in cancer

Tyrosine kinase with immunoglobulin-like and EGF-like domains (TIE), including TIE1 and TIE2 as its principal components, represents a critical group within the receptor tyrosine kinase family. TIE1, specifically, is a transmembrane protein predominantly located in endothelial cells.^378,379^ These receptors demonstrate a notable degree of amino acid similarity in their cytoplasmic domains, while their extracellular regions are less similar but still share some amino acid identity. In the receptors internal structure, there is a split kinase domain that becomes capable of binding to a variety of proteins following self-phosphorylation. The external part of these receptors is characterized by multiple immunoglobulin (Ig)-like domains, dispersed with several EGF-like cysteine repeats and fibronectin type III domains.^380^

The TIE transmembrane protein family, highly receptive to angiopoietins, includes TIE1 and TIE2, with each playing distinct roles in vascular biology. Originally perceived as an orphan receptor, Tie2’s classification evolved following the identification of angiopoietin-1 (Ang1, ANGPT1) as its ligand, along with other ligands such as Ang2, Ang4, and mouse Ang3. Tie2, notable for its broad expression in various cell types including those in larger blood vessels, is especially active during tumor-related angiogenesis.^381^

Advanced structural studies have shown that the extracellular region of Tie2 can form dimers even without ligand binding, facilitated by membrane-proximal fibronectin type III domains. This dimerization process is crucial for its angiogenic functions and its roles in vascular biology, indicating a deeper level of regulatory mechanisms for Tie2 activation and signaling.^382^

Therapies targeting TIE receptors, including monoclonal antibodies against TIE2 and small molecule inhibitors disrupting the TIE-Angiopoietin signaling, are important in the therapeutic landscape. Agents such as Trebananib used in a phase III cancer trial, demonstrated increased progression-free survival in ovarian cancer, highlighting its potential in advanced stages where TIE-1 overexpression is linked to poorer prognosis.^383,384^ Also, The VE-PTP inhibitor AKB-9778 has shown effectiveness in reducing edema and improving vision in retinal vascular diseases, supporting the therapeutic potential of TIE2 activation.^385^

Moreover, Ishibashi et al. have identified a crucial role of TIE-1 in ovarian cancer treatment, specifically regarding cisplatin resistance. Their research clearly separates the functions of TIE-1 from TIE-2, revealing that TIE-1 overexpression correlates with poor prognosis and lower effectiveness of cisplatin in advanced ovarian cancer. This finding suggests the importance of TIE-1 in determining treatment outcomes and highlights its potential as a target for developing new therapeutic strategies in ovarian cancer management.^386^

In a recent study, Marguier et al. demonstrated the significant role of TIE-2 positive (TIE-2^+^) monocytic myeloid-derived suppressor cells (M-MDSC) in melanoma. They found that these cells are more immunosuppressive than their TIE-2-negative counterparts, particularly in advanced stages of melanoma. High levels of TIE-2^+^ M-MDSC were linked to reduced effectiveness of melanoma-specific T-cell responses, with ANGPT2 enhancing the suppressive ability of these cells. The study also indicated that an increased presence of TIE-2^+^ M-MDSC and ANGPT2 in blood is associated with poor prognosis in melanoma. TIE-2 expression on M-MDSC boosts their suppressive features, including the overexpression of inhibitory proteins like PD-L1 and IL-10. ANGPT2 further enhances these immunosuppressive pathways, pointing to the key role of TIE-2 kinase activation in the suppression of T-cell function.^387^

### Deregulation of DDR

Discoidin domain receptors (DDR), specifically DDR1 (CD167a) and DDR2 (CD167b), have been recently recognized as distinctive constituents of the transmembrane RTK family.^388^ These receptors exemplify a distinct subclass of RTKs. In contrast to conventional RTKs, which commonly interact with peptide-like growth factors as their ligands, DDR1 and DDR2 exhibit a distinctive activation mechanism. These receptors are uniquely stimulated through their binding to collagen, which is the most abundant protein found in the extracellular matrix. One important attribute of DDR is their significant participation in the synthesis and degradation processes of collagen, thereby emphasizing their unique function within the RTK family.^389,390^ DDRs contribute not only to the processes of cellular proliferation and differentiation, but also to the dynamics of cell movement, invasion, and attachment.^391^ Moreover, alterations and atypical expression of DDR1 and DDR2 are associated with the advancement of cancer and an unfavorable prognosis.^392–394^

Similar to other RTKs, DDRs are structured into three distinct regions: the outer binding domain, the spanning transmembrane (TM) section, and the inner kinase domain (KD). Both a discoidin (DS) domain and a DS-analogous domain are needed for outer collagen interaction. DDR-connecting proteins bind to TM extracellular juxta-membrane (JM) phosphorylated tyrosines. DDR-connecting proteins bind to phosphorylated tyrosines located in the extracellular juxta-membrane (JM) portion of the TM.^395^ The helical shape of the TM segment allows for receptor pairing without relying on collagen. The receptor’s internal segment consists of the JM zone and the tyrosine kinase KD, which are both essential for its enzymatic activity. Src interaction with tyrosines in the DDR’s collagen-engaged activation loop promotes the phosphorylation process. This series of events has the potential to result in the self-phosphorylation of more tyrosine residues within the kinase domain’s juxta-membrane area. As a result, it attracts adaptor molecules that regulate other cellular processes.^396,397^

DDRs were also associated with the process of EMT, which is influenced by the specific ligand and cell type involved. For example, in prostate cancer cells, the activation of DDR1 triggers the phosphorylation of Pyk2 and MKK7, contributing to the progression of EMT.^398,399^

The invasion of tumor cells depends on matrix metalloproteinases (MMPs), which destroy the extracellular matrix.^400^ For example, DDR1 promotes invasion in MDA-MB-231 BC cells by boosting MMP-2 and MMP-9 secretion.^401^ By increasing MMP-2 levels, DDR1 expression can also cause colon cancer cell invasion.^402^ Recent studies indicate that suppressing DDR2 can decrease B16BL6 melanoma cell invasion by reducing MMP-2 and MMP-9 expression via the ERK/NF-κB pathway.^403^ In contrast, B16-F10 murine melanoma cells with lower DDR2 expression did not affect lung metastasis.^404^

In a study of ESCC, involving mostly male patients (88.3%) with a median age of 68, it was found that high phosphorylated DDR1 (pDDR1) staining, observed in 36.7% of the tumors, is a prognostic indicator. This high pDDR1 staining was significantly associated with shorter recurrence-free and overall survival, highlighting its importance in assessing ESCC progression and prognosis. The study’s findings emphasize the potential role of pDDR1 as a valuable biomarker in understanding and possibly guiding the treatment of ESCC, especially considering the diverse patient characteristics such as age, histological grades, and treatment methods within the cohort.^405^

### Deregulation of LMR

LMR (from lemur tail kinase receptors) are an unusual type of receptors, due to their short extracellular domains and very large intracellular domains, thus their name, include LMR1 encoded by *AATK* gene; LMR2 encoded by *LMTK2* gene and LMR3 encoded by *LMTK3* gene.^406–408^ These membrane-anchored receptors are involved in cell signaling including cell differentiation, invasiveness, migration and proliferation.^409,410^ Even if there are many lemurian tyrosine kinases (LMTKs) studies reported in literature, this family of RTKs is still incompletely characterized. The nomenclature for this kinase family has suffered various modifications along the time: AATYK,^46^ LMR,^411^ LMTK.^408,412^ Some authors consider to not include LMR as a separate family of receptors because of their constitution, being recognized as Ser/Thr RTKs.^409^

With a precise function still to be defined, LMR1 was highlighted as a potential marker for apoptosis, commonly referred to as AATYK (Apoptosis-associated tyrosine kinase), and its overexpression induces differentiation in neuroblastoma SH-SY5Y cells.^407^ Alternative splicing in LMTK1 has given two isoforms LMTK1A and LMTK1B, from which LMTK1A is involved in endosomes recycling process.^409^ Recently, LMTK1 has been reported as a risk factor for Alzheimer’s disease being involved in endosomal localization of amyloid proteins.^413^ LMTK2 is also associated with neurodegenerative dementias,^414^ but no evidence regarding LMR2 has been reported yet. No ligands have been identified for this type of RTKs. LMTK2 and 3 are two of the 27 understudied kinases as stated.^415^

LMTK2 gene expression is affected in the early stages of prostate cancer, this gene could be taken into consideration as a potential biomarker for clinical stratification of prostate cancer patients.^416^ Moreover, LMTK2 gene rs6465657 SNP was detected in the case of prostate cancer in some studies.^416,417^ The LMTK3 gene has been identified as overexpressed in bladder,^418^ breast^419^, and colorectal^420^ cancers. The vast majority of LMTK3 gene mutations are missense mutations, and some somatic mutations were correlated with neuroblastoma.^421^

### Deregulation of STYK1

STYK1 (from Serine/Threonine/Tyrosine kinase) also called NOK (Novel Oncogene with Kinase domain) receptor was reported as an upstream regulator of autophagy.^422^ This receptor that shares homology with PDGFR and FGFR RTKs was reported as tumorigenic and metastatic in nude mice.^423^ Until nowadays no ligand to STYK1 was reported in literature.^424^

STYK1 is involved in metastasis and epithelial to mesenchymal transition and its overexpression is associated with poor prognosis in NSCLC patients.^425^ The ligands for these RTKs family are still unknown.^424^ Overexpression of STYK1 was also noticed in acute leukemia,^426,427^ hepatocellular carcinoma,^428^ ovarian cancer,^429^ NSCLC^430^ and castration resistant prostate cancer patients.^431^ At cellular level, overexpression of this receptor involves cell cycle late mitosis arrest and affects cell division.^432^

Point mutations Y327F and Y356F at the kinase domain might act as tumorigenesis regulators as described by Chen et al in vitro and in vivo studies.^433^ Y417F point mutation at the carboxyl tail plays an autoinhibitory role in modulating signaling transductions.^433^

Deletions in the transmembrane domain regulates the oligomerization function of STYK1 and affects RAS/MAPK signaling.^434^

This receptor is subcellularly found in two different isoforms: dot pattern (DP) and aggregation pattern (AP), from which AP was abundantly found in tumors.^435^ In addition, AP isoform was found to be involved in endocytosis signaling pathways due to its high distribution in endosomes and is considered to have an important implication in intracellular trafficking together with EGFR.^435^

#### RTK signaling nodes, cross-talking, and complementary pathways

RTKs act as critical nodes in cellular signaling networks, intricately linked with several key signaling pathways and regulators. Among the most prominent of these are PTEN, Akt, and mTORC, each playing a distinct yet interconnected role. PTEN serves as a fundamental negative regulator in this network, primarily by dephosphorylating PIP3, thus attenuating PI3K signaling and consequently modulating Akt activity. Akt, a central player in the PI3K pathway, is important for a range of cellular functions, including cell survival, growth, and proliferation. Upon activation by RTKs, Akt phosphorylates a myriad of substrates, leading to diverse cellular outcomes. One of the critical downstream effects of Akt activation is the stimulation of the mTORC1 and mTORC2 complexes. mTORC1, sensitive to nutrient availability and growth factors, regulates protein synthesis and cell growth, while mTORC2 is involved in cytoskeletal organization and cell survival. This cross-talk, facilitated by RTKs, highlights the sophistication of cellular signaling and the potential impact of dysregulation in these pathways, especially in pathological conditions like cancer.^436^

Multiple interconnected pathways are modulated by the RTKs which interact with crucial biological signaling pathways which contribute to critical processes such as survival, cell death mechanisms, proliferation, migration, invasion, and resistance to therapy. FGFR, PDGFR and EGFR are key elements in cellular biology. When binding their ligands, the downstream signaling is triggered and the above-mentioned biological processes are modulated.^437–439^

The RTKs interact with multiple signaling cascades on a large scale. One notable interaction is the cross-talk between RTKs and the WNT signaling which along with interactions with other pathways such as NF-κB and TGF-β, controls homeostasis, cellular differentiation, and developmental processes.

Understanding the complex interconnections among RTK signaling pathways, their cross-talk mechanisms, and complementary pathways is essential for clarifying typical cellular processes and the ways in which dysregulation in these networks plays a role in illnesses such as cancer, immune-related disorders, and developmental disorders. This information forms the foundation for focused therapy approaches meant to change these signaling networks in a way that is therapeutically advantageous.

### RTK-mediated signal transduction and its implications in cellular function and oncogenesis

**Fig. 5:**
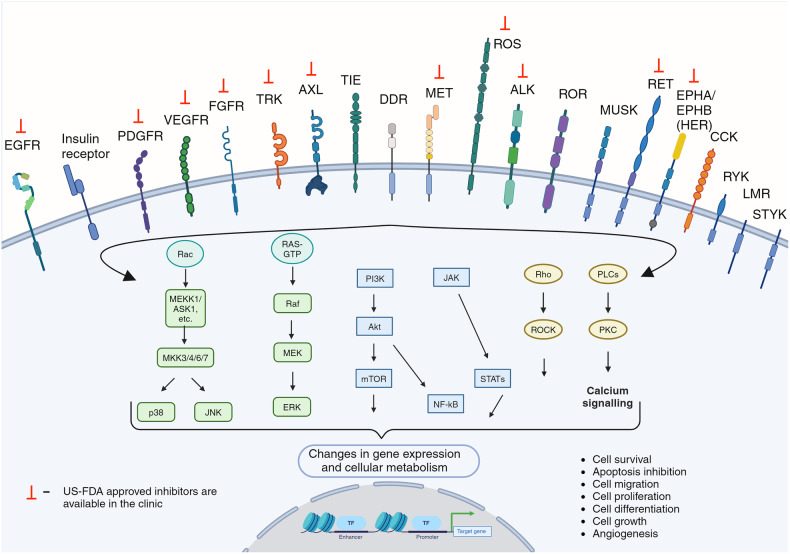
Outline of RTK families—downstream effectors—RTK inhibitors. The RTK downstream signaling is linked to several key biological pathways such as Rac/MEKK/JNK, Ras/Raf/MEK/ERK, PI3K/Akt/mTOR and NF-kB, JAK/STAT or Rho and calcium signaling, modulating gene expression and cellular metabolism. The biological processes that are influenced by the RTKs status are related to cell survival, migration, differentiation, growth, proliferation, and angiogenesis. Images created with BioRender.com

The activation of RTKs triggers multiple signaling pathways, including the Rac/MEKK1/MEKK/JNK pathway, Ras/Raf/MEK1/2/ERL/1/2 pathwayPI3K/Akt/NFKB pathway and JAK/STAT pathway.^440,441^ Multiple molecules were developed to target the downstream effectors of these pathways in order to inhibit tumor cells’ growth and proliferation (Fig. 5).

When binding the EGF, the EGFR is signaling through Ras/Raf/Mek/ERK, JAK/PI3K,STAT, and PKC/NFKB pathways leading to cell proliferation and survival.^442^ The signaling cascade initiated by the IR is focused on the PI3K/PIP3/Akt pathway, which modulates glucose production via FOXO, lipid and protein synthesis via mTORC1, glycogen synthesis via GSK3B and glucose uptake via TBC1D4.^443^ PDGFR signaling modulates Ras/MAPK, JNK/SAPK, PLCγ, PTEN, and Akt/PKB pathways promoting cellular rearrangements, stimulating cell growth, and motility.^444^

VEGFRs are key in the angiogenesis biological pathways and when binding their ligand, the downstream signaling modulates PKC/MEK/ERK1/2, PI3K/Akt, MAPK and Src/FAK pathways, promoting migration, survival, and proliferation.^445,446^Similarly, FGFR signaling is also responsible for survival, differentiation, and proliferation, via signal modulation of RAS/RAF/MAPK/ERK1/2 and PI3K/Akt pathways.^447^

CCK signaling respond to stimuli by modulating Ras/Raf/MEK1/2/ERK1/2, Rac/MEKK/JNK, and P38/MAPKAPK/Hsp27 pathways inducing changes into the actin dynamics, stimulates survival and proliferation of cells.^448^ Survival and apoptosis are also regulated by CD271 (NGFR) via TRAF6/JNK/JUN and RIP2/IRAK/IKK/NFκB pathways.^449^

HFGR/c-MET signaling is important in maintaining survival and increasing proliferation, due to the downstream signaling via Ras/Raf/MAPK, Paxillin/FAK, STAT3/5, and PI3K/Akt/mTOR pathways.^450^

EPHR signaling is very complex, with multiple cross-talks in order to sustain proliferation, differentiation, survival and migration by modulating JNK/STAT, ERK, PI3K/Akt, ABL1/Cyclin-D1, and Ras/ERK pathways.^340,451^ Migration, survival and proliferation are also regulated by the downstream signaling via AXL receptor, through ERK, SRC, P38, and PI3K/Akt pathway, AXL overexpression is correlated with poor overall survival in oncological patients, due to it implication is survival and proliferation.^452^

Besides its role in the tumor microenvironment of metastasis where is directly involved in the blood vessel permeabilization and inflammation,^453^ TIE receptors are signaling through PI3K/Akt pathway modulating cell growth and proliferation. Pathway that is regulated also by PTEN in the interchange between PIP2 and PIP3.^454^

Migration and invasion are key for tumor cells to spread and create metastatic sites, and RTKs are directly involved in these biological processes. When WNT5A binds to RYK receptor initiates the downstream signaling through calcium and through RhoA/ROCK/Akt/ERK/MAPK/P38 inducing an enhanced migration, invasion and inflammation.^455^

DDR1 and DDR2 have key implications in cell survival, growth and adhesion/migration via the downstream signaling through Ras/Raf/MAPK, PI3K/Akt/NFκB, JAK/STAT and Rho pathways.^456^ Rac/JNK, PI3K/Akt and Ras/Raf/MEK/ERK1/2 pathways are also regulated by the RET receptor signaling which is responsible for an enhanced proliferation and differentiation in normal and tumor cells.^457,458^

One of the last remaining orphan receptors, ROS1, are involved in proliferation and survival via signaling through Ras/MEK/ERK, PI3K/Akt and JAK/STAT pathways.^230^ Unlike ROS1, the ROR1 and ROR2 receptors are no longer classified as orphan receptors. Upon ligand binding, they initiate a signaling cascade through the PI3K/Akt/mTOR and Ras/Raf/MAPK pathways, leading to the inactivation of FoxO and the suppression of e-Cadherin. This series of events modulates crucial cellular processes including survival, proliferation, migration, and tumor development.^459–462^

ALK/LTK signaling pathway is responsible for the modulation of cell proliferation, invasion, migration, angiogenesis and apoptosis inhibition, via PI3K/Akt/mTOR, JAK/STAT/VEGF, Ras/Raf/MAPK, and PLCγ/PIP2/IP3 pathways in lymphomas, neuroblastoma and NSCLC.^463,464^

RTKs are crucial in various biological processes such as proliferation, cell survival, angiogenesis, migration, and metastasis formation, via their signaling through key biological pathways. The Ras/RAF/MAPK pathway leads to a cascade of phosphorylation events, which ultimately activate the mitogen-activated protein kinases (MAPKs) which influence proliferation, differentiation, and survival. ERK, the final effector, translocate to the nucleus and further phosphorylates transcription factors leading to gene expression for key pathways for cell development. Another key pathway that is modulated by RTKs is PI3K/Akt/mTOR, which involves the downstream effectors protein kinase B (Akt) and mammalian target of rapamycin (mTOR), regulating metabolism, cell growth and survival. The JAK/STAT pathway is involved in regulating gene expression and immune responses via STAT proteins. RTKs are important in cell signaling by activating these pathways which control the most important cellular processes (Fig. 5). Dysregulations in RTKs or in the downstream effectors can lead to various diseases, including cancer.^17,58,411,465,466^

### Signaling pathways of MAPK in cancer

MAPK pathway is involved in various biological functions such as gene expression, the regulation of blood glucose levels, cell differentiation, tumor progression, drug resistance and survival.^436,467^ The key players in this pathway are RAS, RAF, MEK and ERK1/2 proteins. The cascade begins with Ras, a small GTPase and an upstream protein regulator, which activates Raf. This promotes MEK1/2 followed by the activation of ERK1/2 which regulates different transcription factors, modulating gene expression.^468^ In cancers, this pathway can be activated by cytokine mutations, overexpression of wild/mutant receptors (e.g., EGFR). Moreover, this pathway is important in apoptosis, by phosphorylating apoptosis regulators like Bad, Mcl-1, Bim, caspase-9 or Bcl-2.^469,470^

Ras proteins, encoded by the *HRAS*, *KRAS*, *NRAS*, and additional genes, with several subtypes, including H-Ras, N-Ras, K-Ras, M-Ras or R-Ras, each mediating different pathways within the cell. Specifically, K-Ras is more actively involved in the MEK/ERK pathway, while H-Ras tends to activate the PI3K/Akt pathway. On the other hand, M-Ras and R-Ras also participate in these pathways but are not as frequently mutated in cancers. Mutations in these Ras proteins are detected with different frequencies across cancer types. K-Ras mutations are notably common in a wide array of cancers, whereas N-Ras mutations are predominant only in some cancer.^471,472^

The Raf protein family, which includes A-Raf, B-Raf, and Raf-1, serves as upstream activators of the ERK pathway and also influences apoptosis. Mutations in Raf proteins are frequent in cancers, B-Raf mutations are specific for melanoma, colon cancer, ovary cancer or thyroid cancer (and not limited to these types of cancer). The mutated form of B-Raf is known to overactivate MEK/ERK signaling and can lead to the subsequent activation of Raf-1.^469,473^

MEK1 and MEK2 play a regulatory role in an array of cellular processes, including migration, differentiation, metabolism, proliferation, and apoptosis. Aberrant activation of MEK, particularly through mutation, can decrease the cytokine dependence of hematopoietic stem cells, potentially leading to malignancy.^474,475^

Lastly, ERK1/2 are MAPK superfamily members that mainly modulate apoptosis and proliferation. Activated ERK can phosphorylate various protein kinases in all cellular compartments and can finally lead to the phosphorylation of different transcription factors such as c-Myc, NFκB, c-Jun or Ets-1.^469,476^

#### Mutations in RAS proteins

Mutations in RAS proteins are very common events in tumor development, occurring most frequently at codons 12, 13, and 61. Around 30% of human cancers gain RAS mutations with K-Ras being the most mutated type. In CRC, K-Ras mutations are frequent, particularly in codons 12 and 13, a pattern that is similar to pancreatic cancer. It is hypothesized that K-Ras mutations occur in the early stages of tumor development. In lung cancer, K-Ras mutations are highly prevalent, and may be triggered by epigenetic factors such as chemicals. On the other hand, Ras mutations are rare in hematological malignancies.^477–481^

Several mutations are detected in RAS proteins.^482^ H-Ras displays several point mutations G12V, G12S, G12A, G13D, and Q61R, affecting codons 12, 13, and 61.^483–485^ K-Ras has mutations at codon 12 and 13 (G12D, G12S, G12R, G12A, G12V, G12C, and G13D).^486,487^ Mutations G12D and Q61K are observed in N-Ras and G22V and Q71L in M-Ras.^488–490^

According to Prior et al., five frequent mutations account for 70% of all Ras-mutant proteins (G12D, G12V, G12C, G13D, and Q61R).^489,490^ K-Ras G12C mutation was identified in multiple CRC and lung cancer samples^491–493^ and K-Ras G12D mutation in pancreatic adenocarcinoma^492^ and K-Ras G12D mutation in pancreatic adenocarcinoma.^494–497^

#### Mutations in RAF proteins

RAF MAPK protein is phosphorylated following RAS activation. RAF acts as a mediator for MEK1/2 activation which further modulates ERK. Among the isoforms, B-RAF is the most potent activator of MEK, whereas A-RAF and C-RAF (RAF-1) are less responsive to RAS stimulation compared to oncogenic Src. Both A and C isoforms require RAS-GTP on the cell membrane for activation.^498,499^

As in the case of RAS, RAF mutations are common in cancers like melanoma, CRC, ovary and thyroid malignancies.^500–503^ T1796A mutation in B-RAF gene change valine with glutamic acid in position 600 (V600E) and is the most frequent mutation.^504,505^ Three mutations were identified in C-RAF which induce MAPK cascade by activating C-RAF (G466E, G466V, and G596R).^506^

Smiech et al., synthesized the information related to RAF mutations.^507^ BRAF D594 mutations were identified as follows: D594A in CRC, D594E in melanoma and multiple myeloma, D594G and D594N in NSCLC, multiple myeloma and CRC, and D594H in NSCLC.^508–512^

Three classes of BRAF mutations were identified. Class I—V600E in most of the cancers: V600M in melanoma, skin adenocarcinoma, V600K/R/D in skin cancers. Class II—K601E and G469A in most of the cancers, G469V in lung cancer and lymphoma, G469R in skin cancer and melanoma, G464V in lung cancer and biliary tract cancer, L597Q in CLL, skin cancer and melanoma, K601N in hematological malignancies, L597V in colon cancer and biliary tract cancers, G464E in endometrial carcinoma, and K601T in GCs. Class III—D594N, N581S, G466V, G594G, and D594G in bladder cancer, hematological malignancies, CRC, glioma, pancreatic cancer, lung cancer, melanoma, and head and neck cancers, G466E in BC, skin cancer and melanoma, S467L in skin cancer and melanoma, G469E in oral cancer, skin cancer and melanoma, G466A in lung and skin cancer, G596R in lung and bladder cancer, D594A in liver and colon cancers, D594H in colon and lung cancer, G596D in glioblastoma and F595L in bladder cancers.^507,513–517^ Moreover, the CRAF mutation P261A was identified in lung cancer cell lines and seem to have oncogenic properties, stimulating ERK pathway.^518^

In a cohort of CRC patients assessed by NGS, several RAF mutations were identified, including S467L, R603L, G466V and V600M with different allelic frequencies.^519^ BRAF is mutated in over 60% of melanoma cases, with one single-point mutation accounting for 80% of the BRAF mutations. V600E is activating with 500% more times compared to wild type BRAF.^505,520^

#### Mutations in MEK1/2

The RAS/RAF/MEK/ERK pathway is crucial in tumor growth, with genomic alterations in *RAS* and *RAF* genes that activate MEK to activate a downstream signaling pathway. As a key protein in this pathway, MEK is a promising target for therapies that aim to modulate the pathway and results. MEK is the intermediary between RAS/RAF and ERK. With seven MEK enzymes identified, MEK1 and MEK2 are involved in the RAS/RAF/MEK/ERK pathway, and the primary role of these proteins involves phosphorylation and activation of ERK propagating the signals from receptors such as RTKs to the nucleus. This process regulates gene expression, important for cell survival, differentiation and proliferation.^521–523^

In colon cancer, resistance to MEK inhibitors, often can be due to the presence of specific mutations such as V211D in MEK1.^524^ Moreover, the C121S mutations in MEK1 lead to abnormal kinase activity compared to the wild type. These mutations are classified into three categories based on their RAF interactions. First class of MEK1 mutants with RAF dependent (D67N, P124L, P124S, L177V), the second class of MEK1 mutants, RAF regulated (E203K, L177M, C121S, F53L, K57E, Q56P, K57N, ΔE51-Q58, ΔF53-Q58) and the third class of MEK1 mutants RAF independent (ΔL98-I103, ΔI99-K104, ΔE102-I103, ΔI103-K104), where Δ is indicating a deletion of an amino acids sequence or the fact that the sequence is missing.^525,526^ It was demonstrated that P124L and Q56P mutations in MEK1 confer resistance to MEK and RAF inhibitors in melanomas.^527^

#### Mutations in ERK1/2

ERK1/2 is part of the MAPK pathway, playing a crucial role in cell differentiation, survival and migration. Activated by external stimuli, ERK1 and ERK2 are essential in the RAS/RAF/MEK/ERK signaling cascade. Once activated, ERK1 and ERK2 translocate to the nucleus and phosphorylate transcription factors regulating gene expression. Dysregulations in ERK1/2 can lead to cancer progression, either due to mutations in upstream proteins or overexpression of pathway-triggering receptors, and the result is an uncontrolled cell growth.^528–531^

ERK1/2 is part of the MAPK pathway, playing a crucial role in cell differentiation, survival and migration. ERK1 and ERK2 are activated by extracellular stimuli that are transferred in the downstream cascade via RAS/RAF/MEK/ERK pathway. Once activated, ERK1 and ERK2 translocate to the nucleus and phosphorylate transcription factors regulating gene expression. Dysregulations in ERK1/2 can lead to cancer progression. Even if the dysregulation is a result of mutant upstream proteins or due to overexpression of the receptors that trigger the pathway, the result is an uncontrolled cell growth.^528–531^ERK1/2 mutations can be responsible for the enhanced tumorigenic phenotype of the cells and lead to different cancers.^532^ ERK2 E322K mutation in present in cervical and head and neck cancer.^533–535^

### PAM pathway in cancer

PI3K/Akt/mTOR pathway has a crucial role in cell survival, growth, and cell cycle. Its regulation involves crosstalk with other pathways, and when experiencing abnormalities, the downstream signaling can generate a landscape suitable for malignant cell development. This pathway is frequently activated in cancers and can contribute to resistance to therapy. Dysfunctions in PI3K activity, loss of PTEN or Akt overactivation can lead to cancers and drug resistance.^536–541^

#### Mutations in PI3K and PTEN

PIK3CA is the gene that encodes the p100alpha catalytic subunit of PI3K. Mutations in this gene lead to over-activation of the PAM pathway and uncontrolled cell growth. The D725N and H1047Y mutations in PIK3CA often co-occur with mutations in RAS/RAF pathway.^519^

In a Phase I clinical trial (NCT01219699), hotspot mutations E542K and H1047R/L in PIK3CA were identified in a BC patient. PTEN loss was noted in lung metastasis, while the primary tumor showed E542K and G725G mutations. Metastatic sites exhibited PTEN deletions and mutations S339fs and K342_splice.^542,543^

PTEN is a key tumor suppressor which inhibits cell proliferation and increases sensitivity to apoptosis.^544,545^ Alterations in PTEN function have been identified in a wide spectrum of tumors, indicating that it may control tumorigenesis.^546^ PTEN dysfunction leads to a prolonged PI3K/Akt signaling which induces abnormal cell growth and proliferation.^547^

#### Mutations in AKT

Three isoforms of Akt are encoded by three genes: *AKT1*, *AKT2,* and *AKT3*. AKT1 is involved in survival and antiapoptotic processes, is a key protein in signaling for tissue growth. Due to its involvement in antiapoptotic signaling, it may promote tumor development. *AKT2* signals through the insulin pathway and is involved in glucose transport. While *AKT3* is more expressed in brain and neural tissue.^548–552^

One recurrent mutation in *AKT* is E17K, present in all three isoforms. The *AKT1* E17K mutation is responsible for leukemia development in mice models,^553^ moreover it can induce oncogenic transformation in normal breast epithelial cells (MCF10A).^554,555^ This mutation is present in different types of cancer, with different frequencies, such as BC, endometrial cancer, skin and bone cancer, thyroid and colon cancer.^556–559^

The L52R mutation in *AKT1* was detected in endometrial cancer, and D323G in BC that is ER positive and HER2-negative. In prostate cancer, an AKT2 mutation, L78-Q79ins may be involved in therapeutic sensitivity.^560,561^ In melanomas, E17K and Q79K mutations in *AKT1* increase the resistance to vemurafenib therapy.^562^

#### Mutations in mTOR

The conserved serine/threonine kinase, mTOR, has two distinct protein complexes, mTORC1 and mTORC2 with specific roles in cell signaling. mTORC1 is involved in regulating cell growth, metabolism, and protein synthesis. The downstream effectors S6K and 4E-BP1 are phosphorylated to promote cell proliferation. mTORC2 regulates migration, cytoskeletal reorganization, and cell proliferation. In cancer, dysregulations in mTOR pathway are leading to uncontrolled cell growth and tumor progression. Mutations in the upstream regulators or in mTOR can sustain a prolonged activity contributing to oncogenesis.^563–565^

mTOR mutations were identified in T cells (MOLT16 T cell leukemia cell line), endometrium cells (HEC59, JHUEM7 endometrial carcinoma cell lines) and kidney cells (SNU349 RCC cell line). The mutations C1483Y and R2430M were detected in T cell leukemia, R460*, S2215Y and E1799K in endometrial carcinoma cells, with E1799K also detected in kidney cancer cells.^566^ The mTOR mutation L2209V was identified in a specimen of large cell neuroendocrine carcinoma, and it was showing the ability to transform fibroblasts into tumor-like cells.^567^ The H419R mTOR mutation was detected in thyroid carcinoma cells and G2359E in melanoma tumors.^568^

In RCC, the Y1974H mTOR mutation was identified in a metastatic site in one 57-year-old patient.^569^ According to Ghosh et al., three-point mutations in RCC were identified in mTOR and seem responsible for stimulated cell proliferation (Y1463S, K1452N, A1519T).^570^ H1968Y and P2213S mTOR mutations were described by Kong et al., as gene alterations in melanoma.^571^ L2185A mTOR mutation confers resistance to therapy in colorectal and lung cancers.^572^ In a resistant to treatment thyroid cancer tumor, from a female patient, mutation F2108L was highlighted by Wagle et al., and it appears responsible for the increased resistance to treatment, in conjunction with mutations in other proteins.^573^

### JAK/STAT pathway in cancer

Janus Kinase family includes JAK1, JAK2, JAK3, and TYK2. Janus Kinase/Signal Transducer and Activator of Transcription (JAK/STAT) pathway controls cellular response to signals like growth factors and cytokines. Activated by IFNγ, JAK1, and JAK2 phosphorylate STAT1/STAT3, modulating the inflammation and immunity. JAK1 also phosphorylates STAT5/STAT1, impacting the antigen presentation and antiviral response. STAT3/STAT5 is further involved in mechanisms related to survival, proliferation and angiogenesis.^574–580^

JAK1, JAK2 and TYK2 are ubiquitously expressed, while JAK3 is mostly expressed in hematopoietic cells.^581–585^ TYK2 is implicated in IFN signaling through Toll receptor, mediating the response to LPS.^586^ JAK1 is essential for IL2, IL4, IL15, IL21, and other interleukins, which are also dependent upon JAK3. JAK1 is also key for IL6 and IL11, leukemia inhibitory factor (LIF) and ciliary neurotrophic factor (CNF), INFs and granulocyte colony stimulating factor (G-CSF). On the other hand, JAK2 is essential for hormone-like cytokines such as prolactin, erythropoietin, IL3, IL5, GM-CSF.^587^

The STAT proteins family consists of seven members: STAT1, STAT2, STAT3, STAT4, STAT5A, STAT5B, and STAT6.^588,589^ STAT1 is key for IFNγ signaling and STAT4 for IL12 pathway. Both STAT1 and STAT4 are important factors for Th1 cells polarization,^590^ STAT1 enhancing cell division.^591^ STAT6 modulates IL4 and IL13 via Th2 signaling and may inhibit Th1 polarization.^590,592^ STAT4 can also activate NK cells while STAT5 can promote white blood cells formation, and STAT6 promote B cell proliferation and survival.^591^ STAT3 is excessively activated in AML, multiple myeloma, and various solid tumors like breast, colon, liver, head and neck, lung, and ovarian cancers, correlating with unfavorable clinical outcomes.^593–598^

STAT5 activation has an important role in tumorigenesis. Mutations in STAT5 are few and almost all of them are detected in hematological cancers. When overactivated, STAT5 can enhance the epithelial to mesenchymal transition, induce anti-apoptotic signals and promote invasion and metastases.^599–601^

#### Mutations in STAT proteins

STAT3 and STAT5b are members of the STAT family that are often activated in cancers. The activation of STAT3 can result from upstream kinases, lack of negative regulation, somatic mutations or positive feedback loops. The STAT3 gene is frequently mutated in hematopoietic neoplasms like T-cell large granular lymphocytic leukemia (T-LGL), PTCL, diffuse large B cell lymphomas (DLBCL), anaplastic large T-cell lymphoma (ALCL), and chronic NK lymphoproliferative disorders (NKTCL).^599,602–607^

Mutations in the SH2 domain of STAT3 have been identified across several types of lymphomas: Y640F, D661Y, D661I, D661H, G618R, S614R, and N647I in T-LGL; Q743H in PTCL; D661Y, D661H, N647I, G618R, S614R, and N647I in ALCL; Y640F and I498Y in cutaneous T-cell lymphoma (CTCL); and D661Y, D661H, S614R, and A703T in NKTCL.^599^ Numerous mutations within STAT5b have been observed across various malignancies, including N542H detected in TLGL, NKTCL, and CTCL, Y665F exclusively in TLGL and NKTCL, Q706L in CTCL, T-ALL, and T-PLL. Additionally, unique mutations such as Q743H in PTCL-NOS, T648S in T-ALL, and R659C and Y665H in T-PLL were identified. Notably, all STAT5b mutations were localized within the SH2 domain of the protein.^599^

#### Mutations in JAK1 proteins

Arulogun et al., identified V658I missense mutation in JAK1 in a patient with myeloproliferative neoplasm.^608^ Additional activating mutations of JAK1, including V658F, V658L, and V658I were reported in T cell prolymphocytic leukemia and ALL.^609,610^

Flex et al. evaluated JAK1 mutations in ALL and identified several mutations across different subtypes. In adult B-ALL mutations such as I62V, K204M, A634N and R724H were identified, while no mutations were detected in childhood B-ALL. Moreover, adult T-ALL patients presented eight mutations: I62V, R360W, S512L, A634D, R724H, R879S, R879C and R879H; childhood T-ALL only presented L653F mutation.^611^

Furthermore, in both B- and T-ALL, several JAK1 mutations were described in different protein regions: I62V,^611^ K204M,^611^ R360W,^611^ S512L,^611^ L624_R629>W,^609^ A634D,^611^ S646F,^609^ L653F,^611^ V658F,^609^ R724H,^611^ L783F,^611^ R879C,^611^ R879H,^611^ R879S.^611^ Additionally, V623A and T468S mutations were identified in AML.^612^

#### Mutations in JAK2 proteins

A frequently detected mutation in JAK2 is the V617F missense mutation, commonly found in myeloproliferative neoplasms.^613,614^according to Arulogun et al., 95% of polycythemia vera, 50% of essential thrombocythemia and primary myelofibrosis harbor this mutation.^608^

Haan et al. synthesized multiple mutations in Janus Kinases in hematological disorders.^615^ In megakaryoblastic leukemia, M535I and T875N mutations were identified.^616–618^ Additional JAK2 mutations in polycythemia vera includeF537I,^619^ K539L,^620^ F537-K539delinsL,^620^ H538-K539delinsL,^621^ H538D + K539L + I546S,^622^ H538-K539del,^622^ V536-I546dup,^622^ V536-I546dup11,^621^ F537-I546dup10 + F547L,^621^ I540-E543delinsMK,^621^ R541-E543delinsK,^621^ N542-E543del,^621^ E543-D544del,^621^ D544-L545del,^622^ C618R + V617F.^623^ In B-ALL mutations detected include L611S,^624^ I682F,^609^ I682AQG,^609^ R683G,^609^ R683S,^609^ R683T,^625^ R683K,^626^ R867Q,^609^ D873N^609^ andP933R.^609^

#### Mutations in JAK3 proteins

Recurrent mutations in both JAK1 and JAK3 have been detected in several hematological malignancies by sequencing studies. More than 10% of patients diagnosed with T-ALL have at least one mutation in the *JAK3* gene.^627^

Additionally, one JAK3 mutation A572V was identified in acute megakaryoblastic leukemia.^628^ Among these mutations, several others were detected in the JAK3 protein: P132T,^628^ Q501H + R657Q,^629^ A573V,^630^ M576L,^631^ A593T^631^ and V722I^617^ in acute megakaryoblastic leukemia, while S789P^609^ was detected in childhood B-ALL.

#### Mutations in TYK2

Mutations in TKY2 lead to an upregulation of the downstream STAT signaling.^632^ According to Tomasson et al., G363S mutations were specific for TYK2 proteins in AML.^633^ Multiple mutations were identified in the TYK2 protein, in nonmalignant diseases such as juvenile idiopathic arthritis, rheumatoid arthritis, inflammatory bowel disease, multiple sclerosis or systematic lupus erythematosus and we will not mention them.^634^

## Therapeutic approaches in targeting RTKs

### US Food and Drug Administration (FDA) approved small molecule RTK-inhibitors

RTK-inhibitors are used to treat a variety of malignancies (hematologic, pulmonary, gastrointestinal, endocrinologic and uro-genital cancers) both as first-line therapies such as osimertinib, alectinib or entrectinib and in advanced or metastatic disease such as capmatinib, tepotinib or crizotinib. The FDA-approved inhibitors are either non-selective, targeting multiple RTKs (imatinib and sorafenib), with dual specificity, targeting only 2 RTKs with high specificity (lapatinib and afatinib) or highly selective to only one RTK (gefitinib). Table 11 summarizes the 46 RTK-inhibitors we identified as US FDA-approved by the end of 2023. We specified year of approval; the cancer types the drugs were approved for and the target RTKs. Guidelines and a thorough list of indications are outside the scope of this review; each country and medical system has its own set of guidelines for these cancers.

**Table 11:** Currently US FDA-approved RTK inhibitors

Molecule	RTK-target	Year of FDA approval	Cancer types the drug was approved for
*afatinib*	*EGFR, pan-HER*	*2013*	*NSCLC*
*alectinib*	*ALK*	*2016*	*NSCLC*
*avapritinib*	*c-Kit, PDGFR*	*2020*	*Systemic mastocytosis, GIST*
*axitinib*	*PDGFR, VEGFR, c-Kit*	*2012*	*RCC*
*brigatinib*	*EGFR, ALK*	*2017*	*NSCLC*
*cabozantinib*	*VEGFR, c-Met, RET*	*2012*	*Thyroid cancer, RCC and hepatocellular carcinoma*
*capivasertib*	*pan-AKT*	*2023*	*hormone receptor–positive, EGFR–negative advanced or metastatic BC*
*capmatinib*	*c-Met*	*2020*	*NSCLC*
*ceritinib*	*ALK, ROS1*,	*2017*	*NSCLC*
*crizotinib*	*c-Met, VEGFR, RET, AXL, ALK, ROS1*	*2022*	*NSCLC*
*dacomitinib*	*EGFR, pan-HER*	*2018*	*NSCLC*
*dasatinib*	*PDGFR*	*2006*	*Acute lymphoblastic leukemia, chronic myeloid leukemia*
*entrectinib*	*TRK, ROS1, ALK*	*2019*	*NTRK-mutated solid tumors*
*erdafitinib*	*FGFR*	*2019*	*Urothelial carcinoma*
*erlotinib*	*EGFR*	*2004*	*NSCLC*
*futibatinib*	*FGFR*	*2022*	*Cholangiocarcinoma*
*fruquintinib*	*VEGFR*	*2023*	*CRC*
*gefitinib*	*EGFR*	*2003*	*NSCLC*
*imatinib*	*PDGFR, VEGFR*	*2001*	*Chronic myeloid leukemia, acute lymphoblastic leukemia, dermatofibrosarcoma tuberans, GIST*
*infigratinib*	*FGFR*	*2021*	*Cholangiocarcinoma*
*lapatinib*	*EGFR, HER2*	*2007*	*BC*
*larotrectinib*	*TRK*	*2018*	*NTRK-mutated solid tumors*
*lenvatinib*	*VEGFR*	*2015*	*Thyroid cancer, hepatocellular carcinoma*
*lorlatinib*	*ALK, ROS1*	*2021*	*NSCLC*
*lucitanib*	*VEGFR, FGFR*	*2023*	*BC*
*midostaurin*	*FLT3*	*2017*	*AML*
*mobocertinib*	*EGFR*	*2021*	*NSCLC*
*neratinib*	*EGFR, HER2, HER4*	*2017*	*BC*
*nilotinib*	*PDGFR, EphA4*	*2007*	*Chronic myeloid leukemia*
*osimertinib*	*EGFR*	*2015*	*NSCLC*
*pacritinib*	*FLT3*	*2022*	*Myelofibrosis*
*pazopanib*	*VEGFR, PDGFR, c-Kit*	*2012*	*Hepatocellular carcinoma*
*pemigatinib*	*FGFR*	*2020*	*Cholangiocarcinoma, myeloid/lymphoid neoplasms*
*pexidartinib*	*CSF1R*	*2019*	*Tenosynovial giant cell tumors*
*ponatinib*	*PDGFR, VEGFR, FGFR, Src*	*2012*	*Acute lymphoblastic leukemia, chronic myeloid leukemia*
*pralsetinib*	*RET*	*2020*	*NSCLC*
*quizartinib*	*FLT3*	*2021*	*AML*
*regorafenib*	*VEGFR, PDGFR, c-Kit, FGFR*	*2015*	*Colorectal tumor, GIST, hepatocellular carcinoma*
*repotrectinib*	*ROS1*	*2023*	*NSCLC*
*ripretinib*	*c-Kit, PDGFR*	*2020*	*GIST*
*selpercatinib*	*RET*	*2020*	*NSCLC, RET-fusion-positive solid cancers*
*sorafenib*	*PDGFR, VEGFR, c-Kit*	*2005*	*Hepatocellular and RCC, differentiated thyroid cancers*
*sunitinib*	*PDGFR, VEGFR, FLT3*	*2006*	*RCC, GIST, pancreatic neuroendocrine tumor*
*tepotinib*	*c-Met*	*2021*	*NSCLC*
*tivozanib*	*VEGFR, PDGFR, c-Met, c-Kit*	*2021*	*RCC*
*tucatinib*	*HER2*	*2020*	*BC, CRC*
*vandetanib*	*VEGFR, EGFR, RET*	*2011*	*medullary thyroid cancer*

### Limitations of current RTK-inhibitor therapies

A variety of factors have to be taken into consideration when choosing RTK-inhibitors: patient characteristics (age, fitness, social and family history, comorbidities, previous lines of treatment, occupation and working habits, presence of risk factors); the characteristics of the tumor (staging and tumor extension, tumor morphology, tumor genetics/genomics); the tumor microenvironment that both directly (by interactions with the tumoral cells) and indirectly (by influencing non-tumoral cells, such as fibroblasts) affects efficacy of RTK-inhibitors; pharmacokinetics and dynamics of the RTK-inhibitor; the presence of treatment-related toxicities; acquisition of primary or secondary drug resistance; cost-efficacy; drug availability; patients’ and physicians’ preferences. Due to the paucity of comprehensive clinical data, selecting the best RTK-inhibitor monotherapy or combination therapy is another constraining factor. These treatment-related limitations will be discussed in detail in the upcoming sections, along with some strategies to help overcome these challenges.

### Drug resistance mechanisms to RTK-inhibitors

Cancer cells may have preexisting genetic mutations or alterations that make them less responsive to the inhibitory effects of RTK-inhibitors. This inherent or natural insensitivity of tumoral cells refers to innate or primary resistance. These mutations may affect the target pathway or activate alternative signaling pathways, allowing the cancer cells to bypass the inhibitory effects of the drug. There are several mutations associated with innate resistance to EGFR-inhibitors, such as the oncogenic RAS mutations. The expression of AXL, for instance, is another example of innate resistance to c-Kit (regorafenib) or ROS1-inhibitors (crizotinib, repotrectinib).^635,636^ A recent study demonstrated that the cell cycle phase is also a regulator of innate sensitivity to RTK-inhibitors. Overexpression of cyclin-D1, a cell cycle regulator, for instance, induced resistance to osimertinib.^637^ Other biological factors, such as the localization of the tumor are also a predictor of innate resistance. Even after rectifying for stage and tumor size, right-sided CRCs, for example, have a worse prognosis and respond less to EGFR inhibitors.^638,639^

Another major limitation of current RTK-inhibitors is that resistance may be also acquired during treatment through various mechanisms, including the development of new mutations, activation of compensatory signaling pathways, or changes in the expression of drug efflux pumps. These changes enable cancer cells to survive and proliferate in the presence of the drug. Understanding the specific molecular mechanisms driving both innate and acquired resistance to RTK inhibitors is crucial for the development of more effective therapies. Ongoing research aims not only to identify crucial resistance mechanisms by multimodal clinical, biochemical, histologic, genetic, epigenetic and genomic approaches but also to identify biomarkers and molecular signatures associated with resistance, which can help guide treatment decisions and the development of novel targeted therapies. It is important to note that resistance mechanisms can vary between different types of cancer and even among individual patients with the same type of tumor. Personalized medicine approaches, such as molecular profiling of tumors, are increasingly used to tailor treatment strategies based on the unique characteristics of each patient’s cancer. Resistance to tyrosine kinase inhibitors has been excellently reviewed by Yang et al.^640^

Thus, in our review we focused on drug resistance pathways specifically to RTK-inhibitors, with a focus on recent findings and novel mechanisms. Table 12 summarizes tumor types and potential mechanisms underlying therapeutic resistance.

**Table 12:** Tumor types and potential mechanisms underlying therapeutic resistance

Tumor type	*Possible resistance mechanisms*
NSCLC	‐EGFR, KRAS, ALK, BRAF mutations^641,653,658,913^ ‐Amplification of MET, KIT or HER2^648,649,654^ ‐Activation of metabolic pathways: AXL, NF-kB, GAs6, ADAM17, NOTCH, P53, PI3K/AKT, RAS-RAF, IGF^655,667–672,682^ ‐Epithelial-mesenchymal transition^673,706^ ‐Histological transformation into small cell lung cancer^704^ ‐Dysregulation of the apoptotic cell death pathway^641^ ‐Epigenetic changes^659–663,665,666^ ‐Overexpression of tumor suppressor genes by non-tumoral regions^687^ ‐Cell cycle aberrations^693^ ‐Autophagy^697,698^ ‐Inhibition of pyroptosis^703^ ‐Modulation of drug uptake and transport^712^ ‐Tumor microenvironment^716–719^
Acute or chronic myeloid leukemia	‐*BCR-ABL* or *FLT3-*mutations^644,914^ ‐Modulation of number of cellular receptors on the surface of tumoral cells^686^ ‐Activation of vascular pathways induced by hypoxia^688,689^ ‐Autophagy^915^ ‐Conformational changes of RTK-inhibitor^708^ ‐Modulation of drug uptake and transport^915^ ‐Activation of metabolic pathways: MAPK/ERK, PI3K/AKT, JAK/STAT^915^ ‐Epigenetic changes^915^ ‐Tumor microenvironment^916^
GIST	‐EGFR, KIT, BRAF or IGF1R-mutations^917,918^ ‐Activation of metabolic pathways, such as the sphyingophospholipid pathway^685^ ‐Tumor microenvironment^919^
CRC	‐KRAS mutations^656,920^ ‐Tumor microenvironment^921^
Hepatocellular carcinoma	‐EGFR-mutations^922^ ‐Activation of the PI3K/AKT, (ERK)/MAPK or JAK/STAT pathways^923^ ‐Activation of vascular pathways induced by hypoxia^688,689^ ‐Autophagy^701^ ‐Tumor microenvironment^924^
Thyroid cancers	‐RET mutations^269^ ‐Epigenetic changes^925^ ‐Activation of metabolic VEGFA/VEGFR1 pathway^926^ ‐Tumor microenvironment^927^
Melanoma	‐BRAF, c-KIT, EGFR, MAPK, NRAS-mutations^928^ ‐Activation of metabolic pathways: PI3K/AKT^929^ ‐Tumor microenvironment^928^
BC	‐HER2-mutations^646^ ‐Activation of metabolic pathways: IGF1, NF-kB^682,684^ ‐Cell cycle aberrations^694^ ‐Dysregulation of the apoptotic cell death pathway^696^ ‐Tumor microenvironment^930^
RCC	‐VEGFR2-mutations^931^ ‐Activation of metabolic pathways: PI3K/AKT, JAK/STAT or VEGF^932^ ‐Modulation of drug uptake and transport^712,932^ ‐Upregulation of immune checkpoints^683^ ‐Epithelial-mesenchymal transition^707^ ‐Epigenetic changes^933^ ‐Tumor microenvironment^934^
Urogenital tract cancers	‐FGFR-mutations^643^ ‐Activation of metabolic pathways: EGF, PI3K/AKT^643^

#### On-target mutations as driver of resistance

We have already covered a number of additional on-target genetic alterations that have been described in previous sections. Studies published in the last 5 years have identified new mutations that promote RTK-inhibitor resistance. For instance, resistance to first-generation EGFR-inhibitors may be associated with *EGFR* point-mutations (T790M, C797S, L792F, V843I).^641^ Many of them, such as the C797S cis mutation, may reduce sensibility to newer generation inhibitors, such as osimertinib too.^642^ FGFR-inhibitors, such as erdafitinib, futibatinib or pemigatinib, may also show decreased efficacy after acquisition of several driver mutations in the *FGFR3* gene (N540K, V553L, V553M, V555L, V555M).^643^ Several *FLT3* mutations, such as the N701K, F691L induce resistance to gilteritinib, quizartinib or both.^644^ The same mechanisms (Y1248H, D1246N) have been observed in the case of c-Met-inhibitors, such as crizotinib or capmatinib.^645^ Double-hit mutations, such as *HER* L869R/T862A; L869R/L755S; or L755S/T862A, reduce susceptibility to HER2-inhibitors, as seen with neratinib.^646^ Chromosomal rearrangements of oncogenes may also cause acquired resistance. For EGFR-inhibitors, most of these oncogenic fusions involve the *ALK, BRAF, FGFR, NTRK, RET,* and *ROS1* genes.^647^ *FGFR2* rearrangements have been shown in pemigatinib-resistant patients.^643^ In order to allow the best possible drug selection and customize personalized therapy, it will be crucial in the future to identify not only oncogenic alterations but their correlation with drug resistance as well.

#### Off-target genetic mutations as driver of resistance

Amplification of the *MET* and *HER2* genes or mutations in the *BIRC5* or *TP53* genes lead to EGFR-inhibitor resistance.^641,648–652^ Mutations of *ALK, EGFR,* and *KRAS*, *ALK* copy-number gains or amplification of *KIT* have been associated with resistance to ALK- or c-Met inhibitors, such as crizotinib or capmatinib.^645,653–656^ Also, *MET* amplification and overexpression was correlated to resistance to ALK-, ROS1, and RET-inhibitors.^657^ *BRAF*-fusion is another mutation reported as a resistance mechanism to osimertinib.^658^

#### Epigenetic changes

**Fig. 6:**
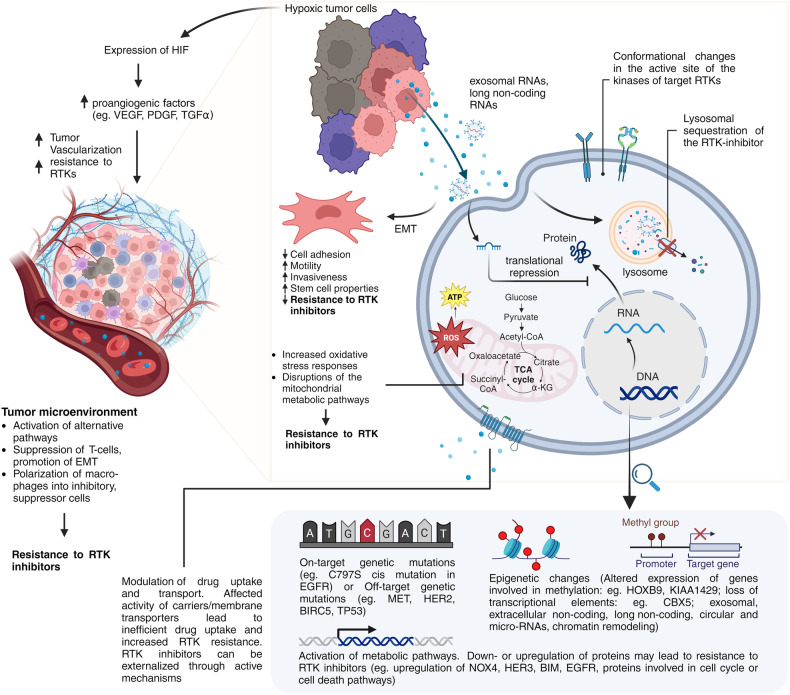
Main resistance mechanisms to RTK inhibitors. The hypoxic tumor cells that express HIF have overstimulated proangiogenic status and promote tumor vascularization and resistance to RTK inhibitors (left). The secreted exosomes containing genetic information (RNAs, long noncoding RNAs) stimulate epithelial to mesenchymal transition disturbing cell adhesion and increasing motility and invasiveness. The stimuli from the hypoxic cells influence other cells behavior by inducing RTKs reorganization, changes in their conformation and protein degradation. Oxidative stress (as an external stimuli) disrupts the mitochondrial metabolic pathways changing the balance within the cells leading to drug resistance. Drug uptake and transport are affected by the changes induced in the carriers and membrane transporters which may disrupt the drug uptake leading to an increased resistance to RTK inhibitors by externalizing the drugs via active mechanisms. The low-right box depicts the resistance to therapy induced by genetic mutations by epigenetic changes that induce mutation in the genes encoding the RTK proteins or other downstream proteins involved in key biological processes such as survival, proliferation or programed cell death. Images created with BioRender.com

Genes involved in DNA methylation, such as *HOXB9* have been correlated to EGFR-inhibitor resistance.^659^ Expression level of epigenetic regulator proteins is also a driving mechanism of RTK-inhibitor resistance (Fig. 6). Gefitinib resistance, for instance, was linked to overexpression of the Vir-like m6A methyltransferase-associated (KIAA1429) protein that regulates methylation processes and synthesis of regulatory RNAs.^660^ Loss of these epigenetic regulators, such as loss of the transcriptional repressor chromobox homolog 5 (CBX5) leads to resistance to EGFR-inhibitors by overexpression of antiapoptotic molecules.^661^ Another novel epigenetic change linked to osimertinib resistance is chromatin remodeling with changes in chromatin accessibility.^662^ Other papers demonstrated that alterations in long-coding RNAs may lead to gefitinib resistance.^663^ Long non-coding RNAs may also play a significant role in RTK-inhibitor resistance by disturbing intercellular communication, the tumor microenvironment, by induction of further epigenetic modifications or by activation of alternative pathways. The role of these RNAs has yet been described for gefitinib, erlotinib, afatinib, and osimertinib, but future research will most likely expand the list beyond EGFR-inhibitors.^664^ Same resistance mechanisms to EGFR-inhibitors have been revealed by exosomes, extracellular non-coding, long non-coding, circular and micro-RNAs.^665^ Mutations in pseudogenes are suggested as possible resistance mechanisms too, such as the expression of *DUXAP10*, which induces resistance to gefitinib.^666^ Targeting RTKs linked to epigenetic regulators may greatly improve therapeutic efficacy and regulate resistance mechanisms. Epigenetics is an important area of research nowadays. Further basic and translational research data are needed.

#### Activation of metabolic pathways

In case of first-generation EGFR-inhibitors (gefitinib, erlotinib), there are several pathways that may lead to resistance when down- or upregulated, such as phosphorylation of Src family kinase, overexpression of the hepatocyte growth factor, activation of the AXL, NF-kB, GAS6, ADAM17, NOTCH, P53, Wnt, PI3K/AKT/mTOR, RAS-RAF pathways.^655,667–672^ JAK/STAT and PI3K/AKT-pathway upregulation, and overexpression of interleukin-17A, IGF1R or FGFR1 seem to play a role in development of resistance to afatinib, a second-generation EGFR-inhibitor.^652,673–676^ Activation and upregulation of the EGFR-pathway, in contrast, leads to adaptive resistance to ALK- and ROS1-inhibitors.^677,678^ Recent studies demonstrated the role of upregulation of NADPH oxidase 4 (NOX4) in resistance to gefitinib and osimertinib.^679^ Upregulation of HER3 was shown to induce resistance to osimertinib.^680^ Upregulation of BIM or EGFR are linked to ALK-inhibitor resistance.^681^ Alternations in the IGF-pathway may lead to several intracellular modifications that eventually lead to decreased sensitivity to EGFR-, c-MET-, and HER2-inhibitors.^682^ Upregulation of immune-checkpoints, such as PD-L1 via the mTOR pathway have been recently associated with development of resistance to VEGFR-inhibitors in RCC.^683^ Urothelial cancers often show resistance to FGFR-inhibitors. One of the main mechanisms revealed is the EGFR and the PI3K/AKT/mTOR pathway bypass activations.^643^ In the case of the HER2-inhibitor lapatinib, a calcium-dependent activation of the RelA (NF-kB) pathway has been observed in resistant cells.^684^ In the case of GIST, the implication of the sphingophospholipid metabolic pathway is suggested to cause resistance to the non-selective inhibitor imatinib.^685^ The number of specific receptors on the surface of tumor cells also influences sensitivity to RTK-inhibitors. For example, the levels of CXCR4 on the surface of acute myeloid leukemia cells regulate responses to the FLT3-inhibitor quizartinib.^686^ Interestingly, overexpression of tumor suppressor proteins may also lead to resistance. In the case of gefitinib, upregulation of CircSETD3, a tumor suppressor in hepatocellular carcinoma, leads to resistance in NSCLC.^687^

#### Activation of vascular pathways

Hypoxia in the tumor microenvironment leads to expression of several hypoxia-induced transcription factors (HIF) that eventually lead to overexpression of downstream proangiogenic factors, such as VEGF, PDGF, or transforming growth factor α. These molecules eventually lead not only to increased tumor vascularization but also to resistance to kinase inhibitors.^688,689^ A switch from angiogenetic to non-angiogenic pathways may induce tumor progression and by activating alternative pathways it may also cause resistance to RTK-inhibitors. There are two main non-angiogenic mechanisms: the vascular co-option, where tumor cells use pre-existing vasculature or the vascular mimicry where vessel-like structures are formed inside the tumor. Both have been demonstrated to cause sunitinib resistance, for instance.^690–692^ The exact mechanisms are not well understood yet. More research on other RTK-inhibitors is required to determine the importance of vascular pathways in the emergence of drug resistance.

#### Cell cycle aberrations

Gene amplifications, especially in the genes encoding cyclin D1, D2, E1, and CDK 4 and 6; and deletions in the CDK inhibitor 2A gene (CDKN2A), are the two main cell-cycle-related changes in cancer causing deregulations in the cell cycle checkpoints.^693^ These mutations may develop during RTK-inhibitor treatment and lead resistance.^647^ Resistance to HER2-inhibitors, for instance, may be caused by cell cycle aberrations. The inhibition of CDK4 and 6 was shown to resensitize BC cells to HER2-inhibition.^694^

#### Modulation of death pathways

Cellular death pathways refer to the intricate and regulated series of events that lead to the demise of a cell. These pathways are fundamental in maintaining the proper functioning and homeostasis of multicellular organisms but may also be implicated in development of cancer and also resistance to RTK-inhibitors. Oxidative stress induced growth inhibitor 1 (OSGIN1) is a regulator of apoptosis, for example. Its overexpression inhibits apoptosis and was shown to induce resistance to gefitinib.^695^ BCL2, another antiapoptotic protein is overexpressed in HER2-inhibitor resistant cells.^696^ Beyond apoptosis regulation, modulation of autophagy may also contribute to resistance. When EGFR is activated, autophagy is inhibited; conversely when EGFR is suppressed, autophagy can be triggered. There is increasing proof linking autophagy to resistance to EGFR-inhibitors. Induction of autophagy may be relevant in development of erlotinib and crizotinib resistance, for instance.^697,698^ Another paper demonstrated the role of improved autophagy in gefitinib desensitization.^699^ However, EGFR T790M mutated cell lines showed increased sensitivity to RTK-inhibitor when these were combined with histone acetyltransferases, that activated the autophagy pathway.^700^ This data suggests that both up- and downregulation of autophagy may play a role in development of resistance to RTK-inhibitors. Autophagy is also involved in resistance to FLT3-inhibitors and sorafenib.^701,702^ Pyroptosis is a form of programmed cell death characterized by a pro-inflammatory response. Unlike apoptosis, which is a non-inflammatory form of cell death. Inhibition of pyroptosis was linked to gefitinib and EGFR-inhibitor resistance.^703^

#### Metabolic reprogramming

Proteomic and transcriptomic data reveal that resistance to TKIs is associated with several metabolic changes: increased oxidative stress responses, hypoxia signatures, disruptions from the mitochondrial metabolic pathways to cytosolic one, such as glycolysis or the pentose phosphate pathway.^704,705^

#### Histological transformation

Epithelial-to-mesenchymal transition (EMT) is one of the mechanisms observed in RTK-inhibitor desensitization. During EMT epithelial cells undergo molecular changes that lead to the acquisition of mesenchymal characteristics leading to reduced cell adhesion, increased motility, invasiveness, and stem cell properties. EMT has been demonstrated to be associated to EGFR- (afatinib) and ALK-inhibitor (crizotinib) resistance.^673,706^ EMT-caused resistance to sunitinib has been demonstrated too.^707^ Inactivation of the tumor suppressors RB1 or TP53 during RTK-inhibitor therapy may result in entire histological shifts, such as from NSCLC to a small-cell subtype.^704^

#### Conformational changes of target RTKs

A novel concept of resistance to highly selective RTK-inhibitors, such as gilteritinib is the conformational changes occurring in the active site of the kinases.^708^ Further studies are required to identify the role of atomistic details behind resistance to tyrosine kinase inhibitors.

#### Lysosomal sequestration of the RTK-inhibitor

There are a few articles reporting the role of lysosomal sequestration of inhibitors in development of resistance by a decrease in the concentration of the drug.^709,710^ A recent paper, however, concluded that these hypotheses cannot be reproduced in vitro, and this mechanism does not actually mediate resistance to imatinib and other RTK-inhibitors.^711^

#### Modulation of drug uptake and transport

Modulation of transporter molecules has also been reported as a metabolic resistance mechanism. Overexpression of solute carrier family 12 member 8 (SLC12A8), an ion transporter, has been demonstrated to play a role in development of resistance of EGFR-inhibitors by activating alternative pathways.^712^ Polymorphisms that affect activity of the ATP-binding cassette (ABC) or solute carrier (SLC) membrane transporters may lead to inefficient drug uptake and RTK-inhibitor resistance.^713,714^ This mechanism has been demonstrated in the case of sunitinib, for instance.^715^

#### Tumor microenvironment

Not only metabolic reprogramming but also reprogramming of the tumor microenvironment mediates resistance to RTK-inhibitors.^716^ Resistance is induced by the microenvironment by multiple mechanisms: activating alternative pathways, suppressing T-cells, promoting EMT or by polarization of macrophages into inhibitory, suppressor cells.^717^ These changes have been observed in EGFR-inhibitor (gefitinib, osimertinib) resistance.^717^ Novel macrophage renewal modes have also been identified, with distinct macrophages contributing to various resistance mechanisms in the same tumor microenvironment.^718^ Extracellular matrix stiffness is another factor contributing to progression and RTK-inhibitor resistance. Upregulation of adaptor-related protein complex 1 subunit sigma 1 (AP1S1), for instance, leads to matrix stiffness and resistance to erlotinib.^719^ Chen et al. extensively reviewed the tumor microenvironmental changes following tyrosine kinase inhibitor therapies, a detailed description of these is out of the scope of our paper.^720^

### Toxicities of RTK-inhibitors

Adverse events can be classified as on-target (due to inhibition of the target receptor) or off-target (due to simultaneous inhibition of multiple kinases), a positive correlation existing between the risk of toxicity and the number of RTKs inhibited.^721^ Thus, a limitation of non-selective RTK-inhibitors is the increased risk for all adverse events. RTK inhibitors, and tyrosine kinase inhibitors, in general, show the highest relative risk (RR = 5.6) of high-grade cardiac toxicity among anti-cancer drugs.^722^ Selective RTK inhibitors that play a major role in development of cardiac manifestations target HER2 and VEGFR.^723^ In contrast with anthracyclines, tyrosine kinase inhibitors were thought to induce cumulative dose-independent, late-onset, non-progressive and partially reversible cardiac toxicities. Recent studies, however, invalidate these theories since several TKIs may cause irreversible cardiac dysfunction.^724^ The mechanism is not well known. HER2-inhibitors, such as lapatinib and afatinib, are mostly associated with heart failure with left ventricular dysfunction and systemic hypertension.^725^ VEGFR (and PDGFR) inhibitors may cause systemic hypertension, the highest incidence being reported with lenvatinib but also observed with cabozantinib, vandetanib and regorafenib; QTc prolongation (axitinib, regorafenib), pulmonary hypertension (lapatinib, lorlatinib) and arterial thrombosis added to the heart failure or systemic hypertension that is associated to all VEGFR inhibitors. Pazopanib was also reported to induce apical ballooning syndrome and fulminant heart failure.^726^ EGFR inhibitors may also induce arterial thrombotic events (erlotinib) or QTc prolongation in case of osimertinib.^727,728^ ALK-inhibitors (crizotinib, ceritinib) and pazopanib have been associated with bradycardia.^722,729^ Non-selective or multi-kinase inhibitors are linked to all beforehand described cardiac events: heart failure (dasatinib), QTc prolongation (nilotinib), right ventricular dysfunction and pulmonary hypertension (dasatinib, bosutinib, ponatinib), accelerated atherosclerosis (ponatinib, nilotinib), increased risk of myocardial infarction and/or cerebrovascular events (ponatinib, nilotinib, sorafenib) and venous thromboembolism.^727,728,730,731^ Pleuro-pericardial effusions are frequently observed with dasatinib, imatinib, ponatinib, bosutinib and FLT3 inhibitors.^732,733^

As a conclusion, cardiac toxicity is a significant concern, as it can limit the use of these drugs, especially in patients with pre-existing heart conditions. All inhibitors may also cause dose-dependent, mild/moderate, reversible diarrhea, constipation, nausea, vomiting, abdominal discomfort, or anorexia. These symptoms are more common in solid tumors compared to the hematologic ones.^734^ Elevated liver enzymes are common in the first months of treatment. These values usually normalize without any intervention. Highest levels have been observed for nilotinib, bosutinib and ponatinib but also for ALK-inhibitors, such as ceritinib and brigatinib.^735^ Skin reactions are one of the most common during RTK-inhibitor therapies. They are usually self-limited. However, dose reduction, drug desensitization or cessation due to severe skin lesions have been reported in the case of erlotinib, afatinib and imatinib.^736,737^ In terms of metabolic and endocrine changes, RTK-inhibitors may cause several electrolytic/glycemic/hormonal imbalances: hyper-/hypo-glycemia (imatinib, sunitinib, nilotinib), hypophosphatemia (imatinib, bosutinib, dasatinib, ponatinib), hypothyroidism (imatinib, sunitinib) or hypogonadism with gynecomastia (sunitinib).^738–740^ Pancreatitis has been reported in a higher incidence in sorafenib-exposed patients.^741^ Pulmonary toxicity may be a significant limitation of RTK-inhibitors since interstitial lung disease (in case of EGFR- and ALK-inhibitors) or drug-induced pneumonitis (following EGFR- or c-MET inhibitors) may be fatal side effects in some patients.^742,743^ Renal adverse effects are usually not life-threatening but severe proteinuria (mostly following VEGFR-inhibitors such as sorafenib, sunitinib, pazopanib), albuminuria (sorafenib, sunitinib) or a transient decline in glomerular filtration rate (imatinib, bosutinib) have been reported.^744,745^ RTK inhibitors used for treatment of chronic myeloid leukemia (due to the simultaneous BCR::ABL-1 inhibitory effects), such as imatinib, bosutinib, dasatinib, nilotinib and ponatinib, may impact blood cell counts, leading to anemia, neutropenia, or thrombocytopenia.^746,747^ This can increase the risk of cardiovascular complications, bleeding, and infections. Because ketoconazole and other antimicrobial drugs may increase plasma levels of RTK-inhibitors, such as sorafenib, regorafenib, lenvatinib, or cabozantinib, while also causing cytopenias, their use may be problematic patient populations.^748^ Another drawback of currently available RTK inhibitors is their usage during pregnancy. Certain drugs are classified as teratogenic (imatinib, erlotinib, lapatinib), while others have not been tested in these circumstances and hence cannot be advised for pregnant women.^749,750^

The application of personalized pharmacogenomics for targeted therapy or drug localization and targeted biodistribution by organ- or tissue-specific nanocarriers may be feasible options in the future to minimize the incidence rate of side effects. Reducing toxicities may also be facilitated by the use of highly selective RTK-inhibitors, as discussed in the paragraphs that follow.

### Approaches to overcome limitations

#### Early detection of relapse/resistance to treatment

Early identification of resistance-driver mutations in circulating tumor DNA (ctDNA) may be an option because switching to another therapy, even before relapse or advancing to a metastatic state may improve outcomes. A recent study demonstrated resistance prediction to ALK-inhibitors in NSCLC patients.^751^ Another study found that an increase in ctDNA levels at the start or during treatment with EGFR inhibitors was associated with resistance and might be used to predict response to therapy in the future.^752^ We found no papers on early prediction of resistance for other RTK inhibitor classes, highlighting the need for additional study in this field.

#### Modulation of pharmacokinetics and biodistribution by nanocarriers

Nanodrug formulations or nanotools as delivery agents, such as liposomal osimertinib, may improve response rates and even overcome resistance because of the increased uptake, targeted distribution, and improvement of therapeutic indexes.^753,754^ Afatinib-loaded nanoparticles showed higher drug concentrations in the tumor tissue, both in lung- and liver cancer patients.^755^ Lapatinib nanoformulations in breast cancer showed higher efficacy due to preferential accumulation in the cancer cells.^756^ Nanoparticles also enable efficient co-delivery of drug combinations, for instance gefitinib in association with cyclosporin A in NSCLC.^757^ The crizotinib-dasatinib combination showed increased permeability through the blood-brain barrier when delivered using micellar formulations in glioblastoma patients.^758^ Nanocarriers may also reduce the incidence of adverse events of RTK-inhibitors, for example imatinib showed significantly decreased cardiotoxicity when loaded in nanoparticles.^759^ There are several other examples of the utility of these novel agents. Smidova et al. excellently reviewed their advantages and current clinical trials on RTK-inhibitor containing nanoparticles.^759^

#### Modulation of downstream or parallel signaling pathways

In case of acquired resistance, targeting alternative oncogenic pathways in combination with RTK-inhibition may overcome resistance mechanisms. Targeting the NOTCH pathway with gamma-secretase inhibitors may increase the efficacy of osimertinib in non-small-cell lung cancer.^670^ Targeting antiapoptotic proteins, such as survivin (BIRC5) in combination with EGFR-inhibitors, may also be a therapeutic opportunity in the future.^661^ In the case of RCC, hypoxia-mediated changes play an important role in the development of resistance. Thus, cabozantinib in combination with hypoxia-induced transcription factor inhibitors is being investigated to inhibit angiogenic vascular pathway activation. Targeting CD70, a marker of EMT, is tested preclinically in EGFR-inhibitor resistant cells.^760^ PROTACs degrade proteins of interest using the endogenous cell proteasome degradation system. PROTAC-mediated targeting of fusion proteins that affect RTK-inhibitor sensibility may give a further treatment option for refractory patients.^761^ Also, in case of autophagy-mediated resistance, Bruton’s tyrosine kinase (BTK) inhibitors seem to efficiently inhibit this resistance mechanism and synergize with FLT3-inhibitors in acute myeloid leukemia.^702^ Another feasible approach would be to combine ALK-inhibitors with PI3Kβ-inhibitors.^762^ Overcoming epigenetic modifications may be feasible by combining RTK-inhibitors with histone deacetylases.^763^ Inhibiting EMT with specific inhibitors in combination with EGFR-inhibitors may also be a feasible option in the future.^764^ Targeting the cell-cycle with CKD-inhibitors combined with osimertinib is now ongoing too (NCT04545710).

#### Adoptive cellular and other targeted therapies

RTK-inhibitor resistant patients may benefit from antibody-based or adoptive treatments, such as chimeric antigen receptor (CAR) T-cells. For example, c-MET targeting CAR-Ts demonstrated efficacy regardless of RTK inhibitor sensitivity.^765^ Antibody-drug conjugates, such as telisotuzumab vedotin which is conjugated with a microtubule inhibitor cytotoxic drug, are other options to target c-Met in combination with EGFR-inhibitors in NSCLC.^766^ CD70, a marker of epithelial-mesenchymal transformation, can be efficiently targeted by CAR T-cells and overcome resistance to EGFR-targeted therapies.^760^ PanErbB-targeting CAR T-cells also showed promising results in head and neck cancer.^767^ Translational researchers should prioritize developing cellular therapeutics for RTK-inhibitor resistant patients, as well as conducting randomized, controlled studies for the agents described above. Combining RTK inhibitors with other non-invasive targeted therapies, such as stereotactic ablative radiotherapy, may improve outcomes, reduce risk of resistance, and even overcome it when present.^768^

#### Discovery of novel tyrosine kinases as therapeutical targets

Discovery of both non-receptor and receptor tyrosine kinases that are associated with resistance is of high clinical impact. Combining RTK-inhibitors with non-receptor tyrosine kinase inhibitors is suggested to improve outcomes in FLT3-mutated acute myeloid leukemia in vitro and in vivo.^769^ But RTK-inhibitor resistance may be also overcome by targeting cytoplasmic tyrosine kinases as well. For instance, protein tyrosine kinase 2 (PTK2) has been shown to be hyperphosphorylated in EGFR-resistant NSCLC patients. Moreover, targeting PTK2 may overcome resistance (10.1186/s12931-019-1244-2). A powerful example for novel RTK targets is EphA2 that has been demonstrated to serve as an escape mechanism in a number of malignancies, including colorectal, breast, liver, and GCs. Because of the documented EphA2-EGFR crosstalk, ALW-II-41-27 is a novel EphA2 small molecule inhibitor that may be crucial in overcoming EGFR-mediated resistance.^770^ ALW-II-41-27 is awaiting clinical trials. Another promising target is AXL. There is no FDA approved selective AXL-inhibitor yet. Bemcentinib, a novel AXL-targeting, highly selective agent demonstrated efficacy and a good tolerability in unfit, chemotherapy ineligible AML patients in the phase 2 NCT02488408 trial.^771^ However, the BERGAMO phase 2 (NCT03824080) trial suggested that monotherapy with bemcentinib offers limited efficacy, a possible combination with hypomethylating agents and venetoclax may be a feasible option in the future.^772^ Promising results have been achieved in NSCLS as well in a phase 1 trial (NCT02922777) but further investigation is needed to determine efficacy of bemcentinib in this context.^773^ A description of RTK-inhibitors that are being studied only in preclinical stages is, however, outside the scope of this article. Our goal is to stress the importance of basic and translational research in identifying novel targets for improving outcomes.

#### Novel-generation RTK-inhibitors

The following paragraphs will address relevant clinical trials assessing RTK-inhibitors that have not yet received US FDA approval, either as monotherapies or, more frequently, in combination with immunotherapies, chemotherapy, or other RTK inhibitors. These novel RTK-inhibitors are summarized below in Table 13.

**Table 13:** Novel RTK-inhibitor molecules recently studied in clinical trials

MOLECULE	TARGET RECEPTOR FAMILY	TRIAL ID OR REFERENCES	CANCER TYPE
*Anlotinib (catequentinib)*	Multi-tyrosine kinase inhibitor	NCT03016819, NCT02586350^777^	Sarcoma, medullary thyroid carcinoma
*Famitinib*	Multi-tyrosine kinase inhibitor	NCT04346381, NCT04129996^812,813^	NSCLC, TNBC
*Foretinib*	Multi-tyrosine kinase inhibitor	NCT00920192^775^	Hepatocellular carcinoma
*Tesevatinib*	Multi-tyrosine kinase inhibitor	NCT02844439 NCT02616393	NSCLC with brain metastases, GBM
*Vatalanib*	Multi-tyrosine kinase inhibitor	NCT00056446 NCT00056459^935^	CRC
*Nintedanib*	Multi-tyrosine kinase inhibitor	NCT02149108 NCT01015118^796,810^	CRC, ovarian cancer
*Sitravatinib*	Multi-tyrosine kinase inhibitor	NCT03906071^820^	NSCLC
*Furmonertinib (Alflutinib)*	EGFR	NCT03787992 NCT03127449 NCT02973763 NCT03452592^781,936,937^	NSCLC
*Aumolertinib (almonertinib)*	EGFR	NCT03849768 NCT04687241 NCT04923906^782^	NSCLC
*Limertinib*	EGFR	NCT03502850^784^	NSCLC
*Abivertinib*	EGFR	NCT03856697^785^	NSCLC
*BLU-945*	EGFR	NCT04862780^804^	NSCLC
*Befotertinib*	EGFR	NCT03861156 NCT04206072^782,938^	NSCLC
*Rezivertinib*	EGFR	NCT03812809 NCT03386955^786,787^	NSCLC
*Sunvozertinib*	EGFR	NCT05712902^788^	NSCLC
*Lazertinib*	EGFR	NCT04248829 NCT02609776 NCT04988295^780,818,819^	NSCLC
*Epitinib*	EGFR	NCT03231501 NCT02590952^789^	GBM NSCLC with brain metastases
*Pyrotinib*	Pan-ErbB	NCT03863223, NCT03588091, NCT03080805^815–817^	HER2 + BC
*Savolitinib (volitinib)*	c-MET	NCT02143466^800^	NSCLC
*Tepotinib*	c-MET	NCT01982955^801^	NSCLC
*Dovitinib*	FGFR	NCT01223027^795^	RCC
*Apatinib (Rivoceranib)*	VEGFR-2	NCT02711007, NCT02824458^806,807,939^	Osteosarcoma, NSCLC, gastric or gastroesophageal adenocarcinoma
*Cediranib*	VEGFR1-3	NCT00777153 NCT00399035 NCT00795340 NCT00245154 NCT00384176 NCT00939848 NCT00532194 (no data yet)	GBM, CRC, NSCLC, biliary tract cancers, ovarian cancer
*Ripretinib*	KIT and PDGFRA	NCT05734105 NCT03353753 NCT03673501^808,809^	GIST
*Surufatinib*	CSF-1R, VEGFR, FGFR	NCT02589821 NCT02588170^792,940^	Neuroendocrine tumors
*Tandutinib*	FLT3	NCT00064584^797^	AML
*Bemcentinib*	AXL	NCT02488408 NCT03824080 NCT02922777^772–774^	AML, NSCLC
*Iruplinalkib*	ALK	NCT03389815 NCT06282536 NCT05351320^791,941^	NSCLC

To attain the lowest possible toxicities and maximum efficacy, novel RTK-inhibitors aim to be highly selective. Nonetheless, there is still ongoing research on novel non-selective or multikinase-targeting drugs, with promising results. Foretinib is one such RTK-inhibitor that has been tested in patients with advanced hepatocellular carcinoma. In the phase 2 NCT00920192 study, these patients achieved a median overall survival of 15.7 months (95% CI 7.9-NR).^774^ Direct comparisons to other agents are awaiting. Preclinical studies suggest that foretinib may be a feasible option as second-line therapy for capmatinib/tepotinib resistant NSCLC patients.^775^ Clinical studies investigating this hypothesis are required. Anlotinib, a Chinese FDA-approved multikinase-inhibitor, is another promising example. It has shown superiority over placebo in trials for medullary thyroid cancer (phase 3 ALTER01031; NCT02586350), synovial sarcoma patients (phase 3 APROMISS; NCT03016819), and NSCLC (phase 3 ALTER0303; NCT02388919).^776–778^

Switching to a newer generation selective inhibitor of the same family may resensitize patients and improve outcomes. The most novel RTK inhibitors we identified target EGFR. One of the most promising, not yet FDA-approved third-generation EGFR-inhibitor, with several phase 3 trials demonstrating its efficacy, is lazertinib. Direct comparison with gefitinib in the LASER301 phase 3 trial (NCT04248829) demonstrated significant superiority in terms of efficacy in NCSLC. Median PFS achieved for lazertinib was 20.6 months (95% CI, 17.8–26.1 months) compared to 9.7 months (95% CI, 9.2–11.3 months) in the gefitinib arm (*P* < 0.001).^779^ Furmonertinib and almonertinib, another third-generation EGFR-inhibitors, also proved to be superior to gefitinib as first line therapy in the phase 3 FURLONG (NCT03787992) and AENEAS (NCT03849768) trials.^780,781^ To compare furmonertinib, almonertinib and lazertinib, additional randomized, controlled trials are needed. Befotertinib was shown to be superior to the first-generation EGFR-inhibitor icotinib in metastatic NSCLC in the phase 3 NCT04206072 trial.^782^ Other EGFR-inhibitors, such as limertinib, abivertinib, rezivertinib and sunvozertinib have been tested in metastatic NSCLC and demonstrated promising efficacy and tolerable safety profile.^783–787^ However, phase 3 trials are required to further assess efficacy. Epitinib is a selective EGFR-inhibitor that proved to be safe and promising in treating GBM and NSCLC with brain metastasis.^788^ Tesevatinib has been studied in both GBM and NSCLC with brain metastasis showing a good permeability through the blood-brain barrier. We identified no phase 3 trials at the moment for tesevatinib and epitinib.

Novel FGFR inhibitors, such as lirafugratinib, were described as promising agents, but they were only studied in a preclinical settings.^789^ Trials are needed to confirm their efficacy in the clinic.

In the case of c-Met inhibitors, switching from crizotinib or capmatinib to the newer generation cabozantinib in NSCLC resensitized patients and led to significantly better outcomes.^645^

In case of ALK-positive NSCLC, iruplinalkib, a new generation ALK-inhibitor showed in the phase 2 INTELLECT trial (NCT04641754) significant antitumoral activity without high-grade toxicities, even in crizotinib-resistant patients.^790^

Another novel target that demonstrated promising results is the CSF-1R-inhibitor surufatinib which significantly improved progression-free survival in both pancreatic and extrapancreatic neuroendocrine tumors in the phase 3 SANET-p (NCT02589821) and SANET-ep (NCT02588170) trial.^791^

Novel generation, selective RET-inhibitors, such as zeteletinib, vepafestinib and TPX-0046 have been reviewed by Clark et al.^792,793^ We identified no phase 2/3 clinical trials on novel RET-inhibitors.

Several RTK-inhibitors demonstrated, however, no improved outcomes or significant toxicities in clinical trials, hence no further studies have been initiated, such as dovitinib for RCC,^794^ nintedanib for CRC,^795^ tandutinib for AML.^796^

#### RTK-inhibitor dual combinations

When resistance to monotherapy is identified, dual/triple drug-combination may improve efficacy. Studies indicate that combining RTK-inhibitors of the same class may help overcome resistance. This strategy, known as vertical pathway inhibition, tries to doubly inhibit the same signaling pathway. These possible combinations include a number of RTK-inhibitors that are not yet FDA-approved. There are several ways of vertical pathway inhibition: combining a multikinase-inhibitor, such as anlotinib, with a highly selective RTK-inhibitor, such as osimertinib, resulted in resistance reversal, for example.^797^ Another approach is targeting an RTK and one of its downstream effectors, such as the RET/mTOR dual inhibition.^798^

EGFR/c-Met dual inhibition by osimertinib and savolitinib was suggested to overcome c-Met resistance in non-small lung cancer patients increasing median PFS from 5.5 (95% CI, 4.1–7.7) to 11.1 months (95% CI, 4.1–22.1).^799^ Other clinical trials (NCT04816214 and NCT03940703) are also examining the combination of osimertinib with other c-Met inhibitors, such capmatinib and tepotinib. Tepotinib was also combined with gefitinib for NSCLC and showed superiority compared to standard-of-care chemotherapy.^800^ In case of lorlatinib (ROS1/ALK-inhibitor) resistance, combination with a pan-HER2-inhibitor (afatinib or dacomitinib) seems to overcome resistance in vitro in NSCLC cells.^801^ Resistance to ROS-1 inhibitor entrectinib may be overcome by adding a MET-inhibitor.^760^ Doublets of third- and fourth-generation EGFR-inhibitors are also studied. The SYMPHONY phase 1/2 trial combined osimertinib with BLU-945, for instance, and achieved less toxicities and promising efficacy.^802^ Drug combinations used in ALK-inhibitor resistant patients have been excellently summarized by Desai et al.^803^ Other studies, such as the phase 3 ACTIVE (NCT02824458) trial, demonstrated that the novel VEGFR-inhibitor apatinib may enhance the effects of EGFR-targeting gefitinib. The combination showed a superior PFS of 13.7 months (95% CI 11.9–14.1 months) compared to a median PFS of 10.2 months (95% CI 10.1–11.9 months) in the placebo + gefitinib arm (*P* = 0.02)^804^ in advanced NSCLC. Tolerable safety profile with promising responses to apatinib have been shown in advanced osteosarcoma patients as well in the phase 2 NCT02711007 trial.^805^

Dual inhibition by the same agent is also possible nowadays, clinical trials studied ripretinib, for example, a dual, highly selective inhibitor of KIT and PDGFRA. Ripretinib significantly improved median PFS, and overall survival compared to placebo in the phase 3 INVICTUS trial.^806^ Although ripretinib did not prove superior to currently used RTK-inhibitor for GIST, sunitinib in terms of efficacy, significantly reduced toxicities were reported in the phase 3 INTRIGUE trial.^807^ A novel phase 3 trial (INSIGHT – NCT05734105) is currently recruiting patients to investigate if efficacy is superior compared to sunitinib in specific genetic subtypes of GIST (NCT05734105). Triple inhibition of VEGFR, PDGFR and FGFR by nintedanib has been studied in NSCLC and CRC. Nintedanib showed promising results in combination with chemotherapy only in ovarian cancer, overall survival did not improve in monotherapy in case of metastatic CRC.^795,808^

#### RTK-inhibitors plus chemoimmunotherapy

Pottier et al. reviewed the effects on immune cells and the immune niche of several RTK-inhibitors, such as cabozantinib, sunitinib, axitinib, imatinib, other FGFR- or VEGFR1-inhibitors. These agents induce overexpression of immune checkpoints, induce proliferation and polarization of anti-tumoral macrophages, induce proliferation of inhibitory regulatory T-cells and produce several anti-inflammatory cytokines.^809^ In light of these, RTK inhibitors are currently being studied in combination with immunotherapies to reduce their immunosuppressive effects and increase efficacy. The non-selective RTK-inhibitor famitinib combined with the novel generation PD-1 checkpoint inhibitor camrelizumab demonstrated high overall response rates (53.7%, 95% CI 37–69%) with a median progression-free survival of 16.6 months (95% CI 8.3-not reached) in advanced NSCLC.^810^ The same combination showed promising antitumor activity in TNBC as well.^811^ Camrelizumab was also combined with apatinib in the phase 1 SPACE study for advanced gastric adenocarcinoma. Although the study sample size is small, the study showed favorable outcomes with a median OS of 17.9 months (95% CI 7.8-not reached) without any surgical intervention.^812^ In case of advanced/metastatic BC, the pan-ErbB inhibitor pyrotinib is being currently studied with trastuzumab, an anti-HER2 monoclonal antibody, and docetaxel as adjuvant-therapy in the phase 3 PHILA (NCT03863223); as neoadjuvant therapy in the PHEBA (NCT03588091) trial or with capecitabine in the phase 3 PHOEBE (NCT03080805) trial.^813–815^

Dual inhibition of RTKs with small molecule inhibitors and mono/bispecific antibodies is an emerging strategy in the clinic. Such a combination is lazertinib combined with amivantamab, an EGFR-MET bispecific antibody, which exhibited outstanding results both with and without chemotherapy in osimertinib-resistant NSCLC patients.^816,817^

However, not in all cases an RTK-inhibitor-immunotherapy combination proved superior to chemotherapy. Such an example is sitravatinib with nivolumab versus docetaxel for NSCLC in the phase 3 SAPPHIRE (NCT03906071) study.^818^

There are several other RTK-inhibitor-chemotherapy combinations that are currently investigated in trials. We are awaiting results. Research questions in the field would be which checkpoint inhibitor to combine with RTK-inhibitors, should we combine more immunotherapies (such as nivolumab plus ipilimumab), should we use classical or novel-generation checkpoint-inhibitors, such as tislelizumab or toripalimab.

## Conclusion and future perspectives

Current research in the field of genomics, proteomics and other related areas facilitated the discovery of various molecular alterations associated with cancer. A significant portion of these alterations have been discovered in RTKs, which impair cellular signaling and contribute to cancer development. The ability to pharmacologically target RTKs due to their functional characteristics has made them a focal point in oncological research. Therapies targeting RTKs have shown remarkable progress in the treatment of cancer, providing survival benefits even for advanced or metastatic cancers. To develop potent and selective inhibitors, researchers and clinicians must have a thorough understanding of the conformation of RTKs and their physiological roles. This allows them to not only aim for better responses but also limit treatment-related toxicities and prevent relapses. The long-term success of these therapies is often held up by the emergence of drug resistance. Ongoing and future research efforts should be concentrated on understanding the molecular basis of these resistance mechanisms. Moreover, it is necessary to conduct research that specifically aims to understand how RTK-inhibitors interact within the cancer microenvironment. This entails developing innovative therapeutic drugs in innovative drug formulations, such as nanocarriers, that can selectively target various RTKs or their subsequent signaling cascades, hence reducing the probability of toxicities and resistance. Personalized approaches, such as analyzing the genetic characteristics of the tumor and tailoring personalized treatment plans, are essential. The objective of these strategies should be to identify patients who are sensitive or primarily resistant to treatment and choose the optimal RTK-inhibitor (combination).

In addition to tumor dynamics, tumor heterogeneity, and other variables, RTK inhibitors exhibit different potency and selectivity within the same class. In the case of BC with brain metastases, for instance, HER2-inhibitors have been compared in a meta-analysis. While the use of lapatinib or tucatinib favored RTK-inhibitor containing regimens, the use of afatinib showed no significant benefit in terms of progression free survival and overall survival.^819^ Afatinib, however, showed significantly longer median survival in NSCLC patients compared to erlotinib or gefitinib.^820^ In RCC, c-Met inhibitors, such as cabozantinib, showed superiority against sunitinib.^821^ However, if cabozantinib is the best currently available option amongst c-Met inhibitors is not well known, since there are no direct comparisons of c-Met inhibitors. Thus, we emphasize the importance of initiation of randomized, controlled studies directly comparing RTK-inhibitors for different cancer types. However, not only efficacy has to be taken into consideration. Several studies showed that nilotinib outperforms dasatinib and imatinib. However, due to financial considerations, imatinib still remains first-line therapy in chronic myeloid leukemia. Other physicians still choose dasatinib instead of nilotinib as second-line therapy. Thus, cost-efficiency, drug availability and physician preferences may also influence which RTK-inhibitor is chosen.

Regardless of the previously mentioned cofounding factors, more molecules must be developed, evaluated, compared, and approved by the FDA in order improve outcomes, reduce toxicities, overcome resistance and maximize the number of patients who benefit from RTK-inhibitors.
